# Degradation-as-signal: a digital-twin framework for disposable optical glucose sensing with lead-free perovskite-inspired films

**DOI:** 10.1039/d6ra01076h

**Published:** 2026-04-22

**Authors:** Erika Vega-Fleitas, Santiago Moll-López

**Affiliations:** a Universitat Politècnica de València Camino de Vera s/n 46022 Valencia Spain ervefl@upv.es; b Departamento de Matemática Aplicada, Universitat Politècnica de València Camino de Vera s/n 46022 Valencia Spain sanmollp@mat.upv.es

## Abstract

Metal-halide perovskites are often regarded as unstable materials whose rapid breakdown under moisture and oxidative stress limits deployment. Here, we invert this paradigm and propose a degradation-driven sensing concept in which controlled chemical decomposition serves as the transduction mechanism for optical glucose detection. We develop a minimal, mechanistically informed, design-oriented digital-twin model for a lead-free halide perovskite-like thin film functionalised with glucose oxidase (GOx). Enzymatic oxidation of glucose produces hydrogen peroxide, which accelerates oxidative degradation of the sensing layer and yields a glucose-dependent loss of optical signal. The framework combines an analytic 0D constant-rate baseline with a GOx-driven transient rate model and a compact power-law optical readout, enabling efficient parameter sweeps over representative glucose ranges (70–300 mg dL^−1^). Simulations predict well-separated decay trajectories and monotonic single-time readouts within practical windows (typically ∼30–45 min). Design optimisation within the explored parameter space identifies an operating point (basal half-life 480 min; accelerated half-life 30 min at 200 mg dL^−1^; *β* = 1.5) yielding a mean classification error ≈1.4% (≈98.6% mean accuracy) across representative clinical pairs and >*rbin*99.9% accuracy for higher–contrast pairs (*e.g.*, 100 *vs.* 200 mg dL^−1^) under 2% additive signal noise. A one-dimensional reaction–diffusion extension for thin films (∼100–500 nm) reveals surface-initiated degradation gradients while preserving a monotonic thickness-averaged signal suitable for macroscopic optical readout. Monte Carlo simulations indicate robust discrimination (≈97–100% accuracy across glucose pairs) under realistic noise. The half-life values explored here should be interpreted as experimentally informed design targets spanning plausible degradation windows, rather than as direct fitted kinetic constants for a single validated glucose-responsive composition. Altogether, the model maps sensitivity, half-life contrast targets, and readout windows, providing quantitative design rules that narrow the experimental search space for lead-free optical biosensors where instability is intentionally harnessed as a functional asset.

## Abstract 2

Controlled degradation becomes the signal: a digital-twin model links GOx-generated H_2_O_2_, lead-free perovskite-like film breakdown and optical decay defining read-out windows for disposable glucose biosensors.

## Graphical abstract

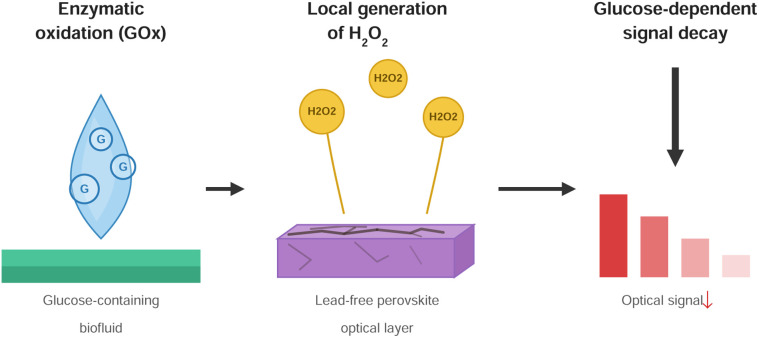

## 1 Introduction

Glucose monitoring is a cornerstone of diabetes management. In 2021, an estimated 529 million people worldwide were living with diabetes, and type 2 diabetes accounted for more than 96% of cases.^1,2^ A substantial fraction of adults with diabetes remain undiagnosed, reinforcing the need for scalable screening and self-monitoring strategies beyond specialist settings.^2^ Clinical guidelines define diagnostic thresholds and risk categories that motivate actionable glucose-range discrimination in practice.^3^ Current technologies—including enzymatic colourimetric assays, electrochemical test strips, and continuous glucose monitoring (CGM) devices—offer high analytical accuracy but often rely on dedicated hardware, specialised electronics, and consumables that require regular replacement.^4^ CGM systems, for instance, rely on subcutaneous enzymatic sensors, on-body electronics and wireless transmitters, and may require calibration workflows, which can limit accessibility and scalability beyond clinical settings.^2,4^

Evidence syntheses indicate that CGM can yield modest improvements in glycaemic control compared with self-monitoring of blood glucose (SMBG), while practical barriers such as cost, standardisation and calibration workflows continue to limit broad deployment.^5–7^ In gestational diabetes, systematic reviews comparing CGM *versus* SMBG report benefits in some glycaemic metrics, but also emphasise heterogeneity across studies and implementation gaps.^6,8–12^ Despite substantial research effort, truly non-invasive glucose monitoring remains challenging; notably, no FDA-approved non-invasive glucose-monitoring device is currently available, in part due to the limited long-term stability of calibration functions required for practical operation.^2^ These constraints have stimulated interest in alternative sensing platforms that are low-cost, disposable, and compatible with decentralised point-of-care or self-monitoring applications.

On the other hand, metal-halide perovskites have emerged as versatile functional materials owing to their strong optical absorption, efficient photoluminescence, and compatibility with low-temperature, solution-based fabrication.^13–18^ Their chemical instability under moisture, oxygen, and oxidising environments is typically viewed as a limitation for photovoltaics and optoelectronics;^14,16,19–22^ however, this same sensitivity has also motivated perovskite-based chemical and environmental sensors, where controlled interaction with water/oxidants produces large readable changes in optical or electrical response.^23^ Recent progress in tin halide perovskites shows that degradation pathways can be chemically tuned and may even display partial ambient *operando* recovery (“self-healing”), highlighting that instability can be engineered rather than merely tolerated.^24,25^

Perovskite-derived signals have also been leveraged for H_2_O_2_ analysis (*e.g.*, electrochemiluminescence platforms), indicating that peroxide-driven interactions can yield quantitative read-outs.^26–31^ For instance, Jia *et al.* reported a perovskite-based electrochemiluminescence (ECL) interface in which H_2_O_2_ catalyses a bio-precipitation process that deposits an insulating precipitate on the electrode surface and quenches the perovskite ECL signal, enabling quantitative peroxide detection.^26^ Building on this sensor perspective, we adopt a different paradigm: material degradation can be used as a transduction mechanism when the chemical trigger is identifiable and controllable.

Here, we investigate perovskite-like films whose degradation is intentionally activated by biochemical products of glucose oxidation. Upon functionalisation with glucose oxidase (GOx), the enzymatic reaction between glucose and oxygen generates hydrogen peroxide (H_2_O_2_), which can accelerate metal-halide perovskite decomposition through oxidative routes.^18,32,33^ The resulting loss of optical signal—photoluminescent or absorbance-based—can therefore be linked to degradation kinetics, enabling a class of disposable biochemical sensors in which the analytical readout is encoded in controlled material breakdown.

Recent large-scale analyses of perovskite ageing further motivate this degradation-based approach. Hartono *et al.* analysed a homogeneous dataset of 2245 maximum-power-point-tracking (MPPT) degradation curves measured under controlled conditions and showed that metal–halide perovskites can exhibit structured, reproducible degradation behaviours that can be clustered, modelled, and correlated with physically meaningful variables such as device architecture and charge-transport limitations.^19^ This supports the use of degradation-oriented reduced-order models as design tools, and reinforces the view that degradation can, in some regimes, be treated as a quantifiable kinetic process rather than as a purely erratic failure mode. At the same time, because degradation trajectories depend strongly on material composition, architecture, and stress conditions, the half-life values explored in the present work should be interpreted as experimentally informed design targets spanning plausible degradation windows, rather than as direct fitted kinetic constants of a single experimentally validated glucose-responsive lead-free composition.

### 1.1 Lead-free candidate materials for degradation-driven optical sensing

Although the modelling framework developed here is intentionally material-agnostic, identifying realistic lead-free candidates is important to ensure that the kinetic regimes explored remain experimentally plausible. For this reason, conventional lead-based perovskites (*e.g.*, MAPbI_3_, FAPbI_3_, CsPbBr_3_) are excluded despite their favourable optoelectronic properties.

A first family of candidates are lead-free double perovskites, particularly Cs_2_AgBiBr_6_. This material is widely regarded as a benchmark lead-free double perovskite because of its high stability, low toxicity, and promising optoelectronic properties.^34^ This compound exhibits strong optical absorption, moderate photoluminescence, and clear environmental sensitivity under moisture and photo-assisted oxidative stress, with degradation products (*e.g.*, AgBr- and BiO_*x*_-rich phases) that align naturally with a degradation-driven transduction concept in which the rate of breakdown encodes glucose concentration. Cs_2_AgBiBr_6_ has also been processed into thick-film and composite architectures suitable for optical devices, including composite CsPbBr_3_/Cs_2_AgBiBr_6_ thick films demonstrating stable visible photodetector operation.^34,35^

A second group comprises bismuth-based halides such as A_3_Bi_2_Br_9_ and A_3_Bi_2_I_9_ (A = Cs, MA, FA), which are perovskite-inspired, solution-processable, and often responsive to humidity and oxidants—features that can be advantageous when instability is intentionally harnessed as a signal.^36^ Finally, tin-based hybrid perovskites (*e.g.*, MASnI_3_, FASnI_3_) exhibit rapid oxidative degradation that could support fast responses, albeit with handling constraints due to air sensitivity;^24,25^ a broader perspective on stability challenges and engineering strategies in tin-based halide perovskites (including nanocrystals) is available in recent studies.^37,38^

A more detailed, literature-grounded discussion of these candidate families (including representative optical response channels and device demonstrations) is provided in the SI (Section S2). In this context, Cs_2_AgBiBr_6_ is used primarily as a plausibility anchor for optical activity, thin-film geometry, and environmental sensitivity, while the numerical framework remains transferable across candidate lead-free chemistries.

### 1.2 Application context and modelling objective

Such degradation-mediated mechanisms are particularly appealing for skin-interfaced and wound-facing applications, where small volumes of biofluids (*e.g.*, sweat, interstitial fluid, or wound exudate) contain glucose and may present oxidising species. Sweat-based glucose monitoring, for instance, has been explored as a minimally invasive alternative to CGM systems.^2,4^ Moreover, chronic diabetic wounds can display elevated glucose together with oxidative biomarkers, suggesting that degradation-driven sensing could be relevant in wound-healing contexts with minimal instrumentation.

To enable rational design of these systems, a quantitative description of how glucose concentration, enzyme-mediated H_2_O_2_ production, perovskite degradation, and optical response are dynamically coupled is essential. The aim of this study is therefore to establish a minimal yet analytically tractable mathematical model capturing: (i) glucose concentration in the surrounding biofluid, (ii) enzymatic generation of H_2_O_2_ by GOx, (iii) oxidative degradation kinetics of a perovskite-like material, and (iv) the resulting temporal evolution of the optical signal. Rather than focusing on a single composition or device architecture, we explore a generic design space for perovskite-inspired systems whose sensing mechanism is rooted in controlled instability.

Concretely, we: (i) develop a reduced-order 0D model that yields closed-form signal trajectories for rapid design sweeps, (ii) introduce a one-dimensional reaction–diffusion extension to assess thickness-driven gradients in thin films, (iii) perform parameter space optimization identifying optimal configurations, (iv) validate discrimination performance *via* Monte Carlo simulation with realistic measurement noise, and (v) quantify separability, half-life contrast targets and candidate read-out windows. These elements provide quantitative design rules (*e.g.*, kinetic contrast, half-life targets and read-out windows) that narrow the experimental search space for lead-free, disposable optical biosensing concepts. Throughout this work, the prescribed half-lives are treated as experimentally informed design targets spanning plausible degradation regimes for lead-free perovskite-inspired films under oxidative or environmental stress. They are not presented as direct fitted kinetic constants for a single already-validated glucose-responsive material platform, but as screening-level targets intended to guide material down-selection and experimental calibration.

**Fig. 1:**
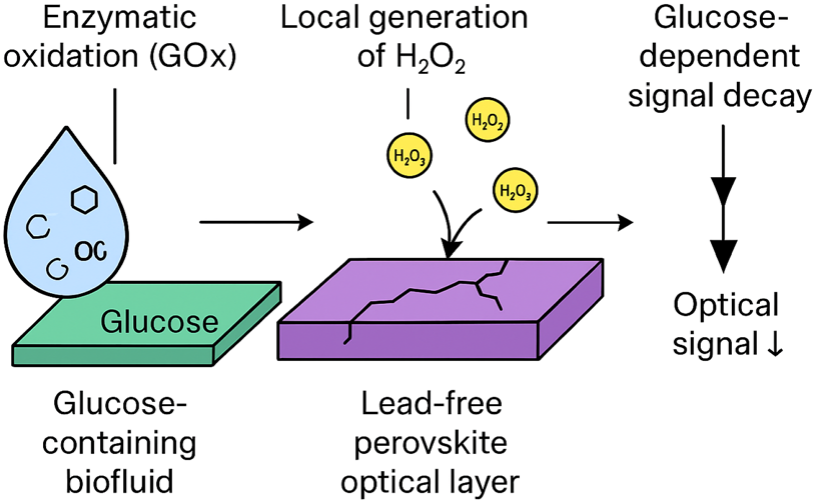
Schematic illustration of the degradation-driven glucose sensing principle. Glucose present in a biofluid is enzymatically oxidised by glucose oxidase (GOx), leading to the local generation of hydrogen peroxide (H_2_O_2_). The peroxide accelerates the oxidative degradation of a lead-free perovskite-like optical layer, resulting in a progressive loss of optical signal. The rate of signal decay encodes the glucose concentration through controlled material breakdown.

Fig. 1 illustrates the sensing principle proposed in this work, where glucose concentration is transduced into an optical signal through controlled, enzyme-mediated degradation of a lead-free perovskite-like layer.

From a broader perspective, the proposed modelling framework constitutes a material-level digital twin of the sensing layer. Here, the digital twin is defined as a reduced-order, mechanistically informed computational surrogate that links biochemical input (glucose concentration), internal material state (degree of degradation), and observable output (optical signal). Rather than aiming at real-time device mirroring, the model is used as a design and optimisation tool, enabling virtual exploration of kinetic regimes, sensitivity limits, and read-out strategies prior to fabrication. In this way, the framework shifts material degradation from an undesired ageing effect to a quantifiable sensing feature within a controlled operating window.

## 2 Model and methods

This work develops a minimal numerical model to assess the feasibility of a degradation-driven optical sensor for glucose detection based on a perovskite-like material. The objective is to capture, using a transparent formulation with few parameters, the essential couplings between: (i) glucose concentration, (ii) enzymatic production of hydrogen peroxide (H_2_O_2_), (iii) degradation of the sensing layer, and (iv) the resulting optical signal. This section summarises the model assumptions, governing equations, and the simulation workflow.

### 2.1 Conceptual overview

The proposed sensor comprises a thin perovskite-like layer that undergoes chemical degradation in the presence of H_2_O_2_. To impart glucose sensitivity, the layer is assumed to be functionalised with glucose oxidase (GOx), which catalyses glucose oxidation and generates H_2_O_2_ as a by-product. Accumulated peroxide increases the effective degradation rate of the film, which in turn reduces an optical observable (*e.g.*, photoluminescence or absorbance). The sensor response is therefore encoded in the time evolution of degradation-driven signal loss.

The model relies on three core assumptions:

(1) GOx-catalysed glucose oxidation operates in a quasi-steady kinetic regime over the time scales of film degradation, enabling a lumped mapping from glucose concentration to peroxide availability.

(2) Film degradation is described by pseudo-first-order kinetics with respect to the fraction of intact material.

(3) The optical signal is a monotonic function of the remaining intact fraction.

These assumptions yield an analytically tractable baseline model that captures first-order sensor behaviour while limiting parameter degeneracy and preserving interpretability.

#### 2.1.1 Relationship between the reduced-order and transport-aware models

We use a two-tier modelling strategy. First, a reduced-order formulation provides a fast, analytically transparent screening tool that links glucose-driven oxidative stress to an effective degradation rate and to the resulting optical signal decay. Second, an optional one-dimensional extension is introduced to test whether finite film thickness and reactive-species transport can generate internal gradients that deviate from the spatially homogeneous assumption. In this way, the reduced-order model supports rapid exploration of read-out windows and kinetic contrast, whereas the transport-aware model is reserved for geometry-specific validation when thickness-dependent effects may matter. This distinction becomes particularly relevant when transport resistance, barrier layers, or porous morphologies are expected to shift the system away from the spatially homogeneous limit.

### 2.2 Materials selection

The modelling framework is intentionally material-agnostic; nevertheless, lead-free composition is treated as a primary constraint for the envisioned disposable, skin-interfaced formats. Candidate families (lead-free double perovskites such as Cs_2_AgBiBr_6_, Bi-based halide derivatives such as A_3_Bi_2_Br_9_, and optional high-reactivity Sn-based hybrids) are introduced in Section 1.1, with a literature-grounded discussion provided in the SI (Section S1 and S2). In the present work, material variability is represented through prescribed half-lives and the optical nonlinearity parameter *β*, enabling transferable design sweeps without committing to a single composition.

### 2.3 Production of hydrogen peroxide

The sensing mechanism relies on enzymatic glucose oxidation catalysed by glucose oxidase (GOx), during which glucose is converted into gluconolactone with stoichiometric production of hydrogen peroxide (H_2_O_2_).^39,40^ The reaction rate is described by Michaelis–Menten kinetics,1
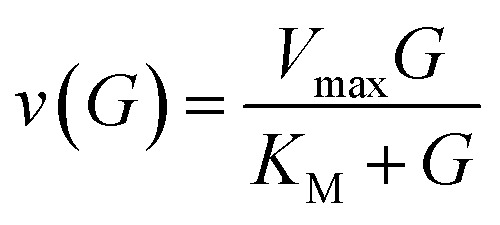
where *V*_max_ is the maximum turnover rate, *K*_M_ is the Michaelis constant, and *G* is the local glucose concentration in the surrounding medium (expressed in mM). In this expression, *v*(*G*) represents the instantaneous rate of H_2_O_2_ generation. GOx is particularly attractive for biosensing because of its high specificity, high turnover, and high stability.^40^

Throughout the governing equations, glucose concentration *G* is expressed in millimolar (mM), consistent with the Michaelis–Menten parameters used for GOx kinetics. For clinical interpretability, concentrations are occasionally reported in mg dL^−1^; the conversion for glucose (molar mass 180.16 g mol^−1^) is:2
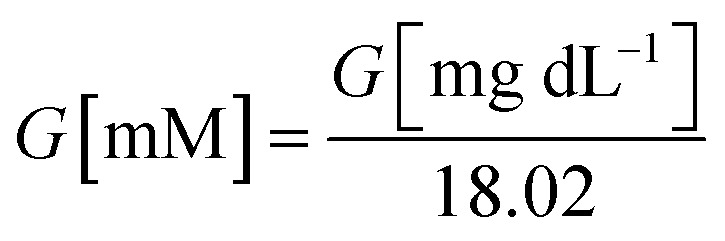


Reported *K*_M_ values for immobilised GOx often lie above glucose concentrations encountered in sweat, interstitial fluid or wound exudate.^39–46^ For example, GOx covalently bonded on pHEMA membranes has been reported with *K*_M_ ≈ 8.8 mM and *V*_max_ ≈ 0.067 mM min^−1^, which are of the same order as the representative values adopted here for screening-level simulations.^47^ Under conditions where *G* ≪ *K*_M_, eqn (1) reduces to the linear regime,3
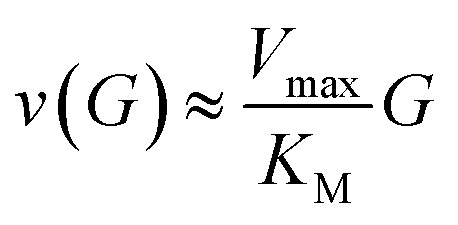


To keep the model reduced-order, we adopt a lumped approximation in which the effective peroxide availability at the sensing layer scales with glucose:

4 [H_2_O_2_]_eff_ = *αG*

where *α* aggregates enzyme loading, immobilisation efficiency, oxygen availability, hydration state, and transport/retention effects (including peroxide diffusion and consumption).

Representative values of *K*_M_ and *V*_max_ for immobilised GOx depend on enzyme loading, immobilisation matrix, temperature, pH, oxygen access, and local hydration. Accordingly, the values used in the present simulations are intended as literature-informed near-ambient, near-physiological representative parameters rather than universal constants. The linear mapping in eqn (4) should therefore be understood as a reduced-order screening approximation; when glucose levels, immobilisation conditions, or oxygen transport place the system closer to enzymatic saturation, the full Michaelis–Menten production term should be retained. Sensitivity of the response to this approximation is discussed in the SI (Fig. S4 and S5).

### 2.4 Degradation kinetics of the sensing layer

Let *C*(*t*) denote the fraction of intact material at time *t*, with *C*(0) = 1. Film degradation is modelled as a pseudo-first-order process:5
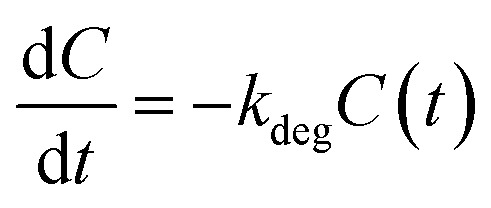
where *k*_deg_ is an effective degradation rate constant. We write

6 *k*_deg_ = *k*_0_ + *k*_1_[H_2_O_2_]_eff_ = *k*_0_ + *k*_1_*αG*

where *k*_0_ captures basal degradation pathways and *k*_1_[H_2_O_2_]_eff_ captures peroxide-accelerated degradation. For compactness, we define *k*_*G*_≡*k*_1_*α*, so that *k*_deg_ = *k*_0_ + *k*_*G*_*G*.

Within the reduced-order formulation, *k*_1_ and *α* are not independently identifiable because they enter only through the product *k*_*G*_ = *k*_1_*α*. Experimentally, this means that enzyme loading, immobilisation efficiency, local peroxide retention, and related microenvironmental effects are observed through the net glucose-to-degradation coupling *k*_*G*_, which is the quantity directly accessible during calibration.

Substituting into eqn (5) yields

7 *C*(*t*) = exp[−(*k*_0_ + *k*_*G*_*G*)*t*]

linking glucose concentration to the decay rate of the intact fraction.

### 2.5 Optical signal model

The measured optical signal *S*(*t*) is assumed to correlate with the structural and optoelectronic integrity of the sensing layer. We use the compact phenomenological relation

8 *S*(*t*) = *S*_0_*C*(*t*)^*β*^

where *S*_0_ is the initial signal and *β* is an empirical exponent capturing nonlinearities between intact fraction and optical output.

### 2.6 Model parameterisation from prescribed half-lives

To connect the reduced-order model to experimentally plausible time scales, we parameterise the degradation rates using prescribed half-lives treated as design targets rather than direct fitted constants of a single validated material platform. At present, the literature does not provide directly measured glucose-coupled or H_2_O_2_-coupled degradation half-lives for lead-free halide perovskite thin films in biosensing architectures of the type considered here. Published studies on candidate lead-free materials, including Cs_2_AgBiBr_6_, instead provide qualitative evidence of distinct stability regimes—for example, high intrinsic stability under benign ambient conditions together with substantially faster degradation under moisture and photo-assisted stress.^48^ Accordingly, the nominal half-life values used in this work are not presented as literature-fitted material constants, but as screening-level design targets that probe whether a practically useful discrimination window could exist between a slow basal regime and an analyte-accelerated regime. For a pseudo-first-order process, *t*_1/2_ = ln  2/*k*_deg_.

In the absence of glucose (*G* = 0):9
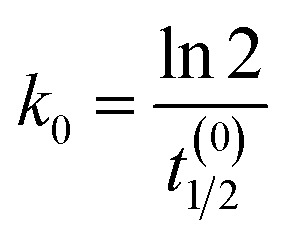


At reference glucose *G*_ref_:10
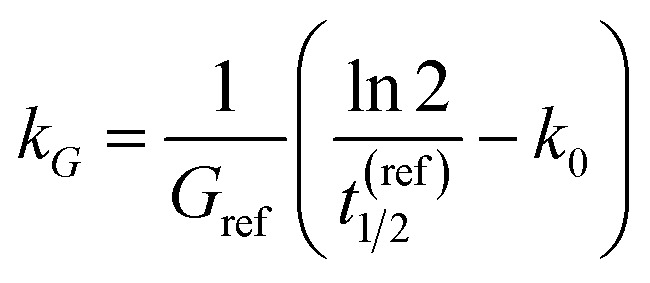


### 2.7 One-dimensional surface-driven peroxide transport and local degradation

To evaluate whether finite film thickness can induce spatially heterogeneous degradation, we extend the reduced-order model by introducing an effective hydrogen-peroxide concentration field *H*(*x*, *t*) (mM) across a film of thickness *L*, with *x* ∈ [0, *L*] measured from the exposed surface. The governing equation reads11
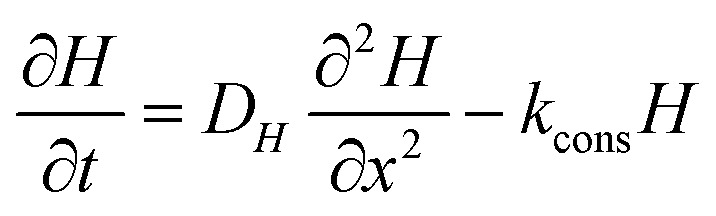
where *D*_*H*_ is an effective diffusion coefficient and *k*_cons_ is an effective peroxide-consumption rate constant. This choice is consistent with the fact that hydrogen peroxide transport through hydrated polymeric or membrane-like environments can be substantially hindered relative to free aqueous diffusion, so *D*_*H*_ is interpreted here as an effective in-film transport coefficient rather than a bulk-solution value.^49^

The exposed surface is driven by a time-dependent Dirichlet boundary condition,

12 *H*(0, *t*) = *H*_*s*_(*t*; *G*)

where *H*_*s*_(*t*; *G*) is the peroxide level generated by GOx kinetics:13
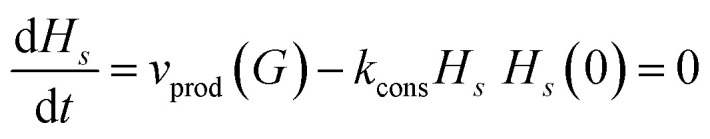
with Michaelis–Menten production rate14
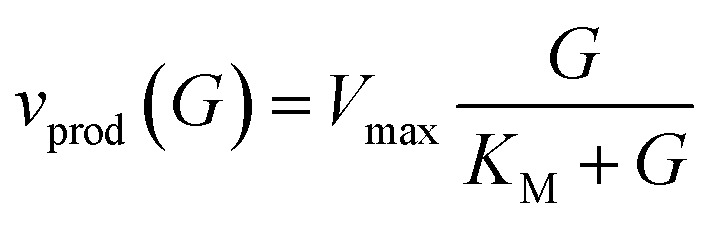


At the film/substrate interface:15
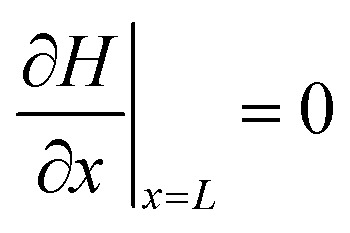


Local integrity *C*(*x*, *t*) evolves *via*16
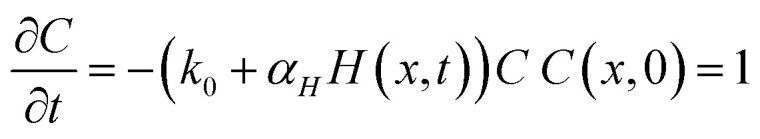
and the optical read-out is

17 *S*(*x*, *t*) = *C*(*x*, *t*)^*β*^

with thickness-averaged signal18
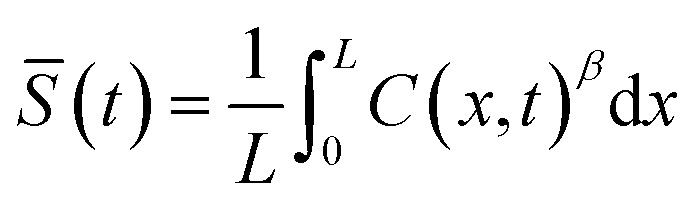


### 2.8 Computational implementation and numerical validation

The reduced-order (0D) constant-*k* model is evaluated directly from the analytical expressions in eqn (7) and (8), enabling rapid parameter sweeps and closed-form benchmarking. For the GOx-mediated variant, the peroxide build-up and integrity dynamics are integrated numerically using a standard adaptive explicit Runge–Kutta ODE solver from SciPy with relative and absolute tolerances set to 10^−6^. This numerical layer is only required for the time-dependent peroxide pathway; all constant-*k* trajectories remain analytical.

For the one-dimensional (1D) reaction–diffusion extension, the peroxide transport equation (eqn (11)) is discretised using second-order centred finite differences in space and explicit forward Euler time stepping. The time step Δ*t* is selected automatically to satisfy the classical diffusion stability condition Δ*t* ≤ Δ*x*^2^/(2*D*_*H*_) with a safety factor of 0.5, ensuring numerical stability across the explored thickness range. The Dirichlet boundary condition at the exposed surface is imposed directly, whereas the Neumann condition at the substrate interface is implemented with a ghost-point construction. Local integrity *C*(*x*, *t*) is updated pointwise using the exact exponential solution over each Δ*t*, which improves robustness relative to a purely explicit update for stiff kinetic regimes.

To ensure internal consistency between modelling tiers, we implement sanity checks that compare the thickness-averaged 1D signal *S̄*(*t*) to the corresponding reduced-order baseline under matched conditions. Specifically, we report (i) the end-point difference in *S̄*(*t*) at the final simulated time, and (ii) the root-mean-square (RMS) deviation over the full trajectory. Importantly, when the GOx-mediated pathway is re-parameterised to match a chosen reduced-order target, the end-point agreement can be made arbitrarily close while transient differences remain due to peroxide build-up dynamics and transport-induced heterogeneity. All figures are generated at 300 DPI using Matplotlib. A consolidated summary of model parameters, units, representative values, and modelling roles (fixed, calibrated, or design variables) is provided in Table S1 of the SI.

### 2.9 Dimensionless formulation and universal degradation behaviour

Defining dimensionless time *τ* = *k*_0_*t* and sensitivity parameter *g* = (*k*_*G*_*G*)/*k*_0_, eqn (7) becomes

19 *C*(*τ*) = exp[−(1 + *g*)*τ*]

This representation shows that all material-, enzyme- and environment-specific effects enter the reduced-order model through the single ratio *g*, clarifying transferability across candidate compounds and architectures.

## 3 Results

### 3.1 0D degradation dynamics and glucose-dependent response

We first evaluate the reduced-order (0D, spatially homogeneous) formulation in which glucose modulates the effective degradation rate through *k*_deg_(*G*) = *k*_0_ + *k*_*G*_*G* (Section 2.4), with kinetic constants parameterised from prescribed half-lives (Section 2.6). For clarity, glucose concentrations are reported in mM; the commonly used clinical unit (mg dL^−1^) is provided in parentheses using eqn (2). In particular, 3.9, 5.6 and 7.0 mM correspond approximately to 70, 100 and 126 mg dL^−1^, respectively.

**Fig. 2:**
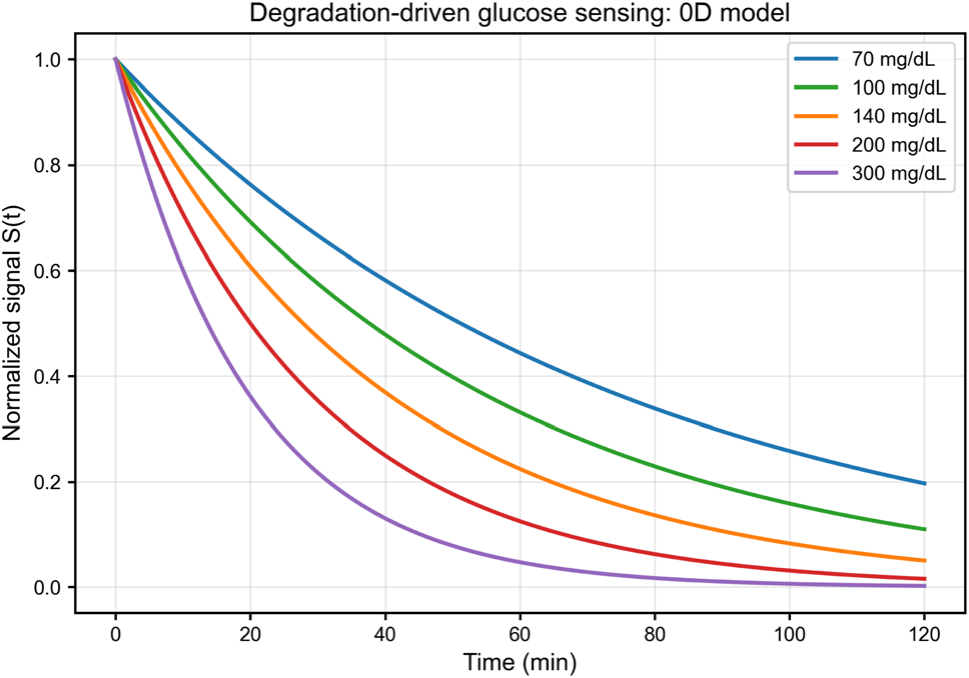
Temporal evolution of the normalised optical signal *S*(*t*) for glucose concentrations of 3.9–16.6 mM (70–300 mg dL^−1^) under the 0D model. Higher glucose produces a larger effective degradation rate and hence faster signal decay, establishing monotonic kinetic contrast. Trajectories are color-coded by clinical glucose range (blue: hypoglycemic, green: normoglycemic, orange: prediabetic, red–purple: diabetic).

Fig. 2 shows the temporal evolution of the normalised signal *S*(*t*) predicted by the 0D model for glucose levels ranging from 70 to 300 mg dL^−1^. Increasing glucose increases *k*_deg_(*G*), leading to faster signal decay and a consistent ordering of trajectories across the explored range. This monotonic kinetic contrast constitutes the core transduction principle of the proposed degradation-as-signal mechanism. Half-lives range from approximately 77 min at 70 mg dL^−1^ to 20 min at 300 mg dL^−1^, providing substantial kinetic contrast over clinically relevant glucose ranges.

### 3.2 Single-time read-out response

**Fig. 3:**
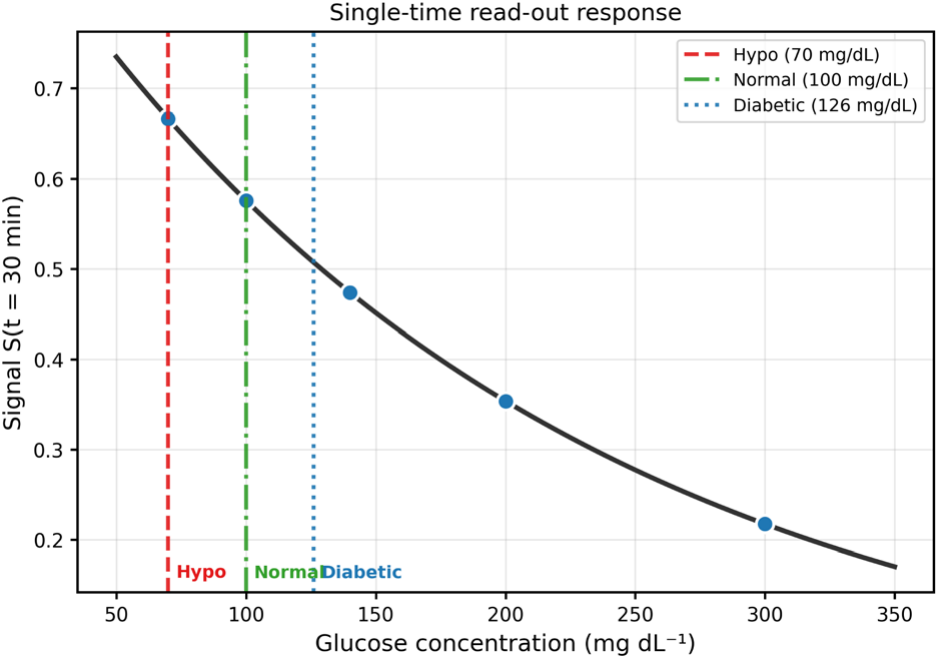
Normalised optical signal at *t*_read_ = 30 min as a function of glucose concentration in the 0D model. The response is monotonic over the explored range for the chosen half-life parameterisation, supporting the use of a single-time read-out as a practical operating mode. Markers indicate discrete measurement points; the curve represents the continuous analytical solution. Vertical dashed lines indicate clinical thresholds at 3.9 mM (70 mg dL^−1^), 5.6 mM (100 mg dL^−1^), and 7.0 mM (126 mg dL^−1^).

To emulate a practical single-shot measurement, we consider a fixed read-out time *t*_read_ = 30 min. Fig. 3 reports *S*(*t*_read_) as a function of glucose concentration in the 0D model, exhibiting a monotonic decrease over the explored range for the adopted half-life parameterisation. This mapping supports the use of a one-time optical read-out as a practical operating mode. Its robustness with respect to optical nonlinearity and kinetic uncertainty is further examined in the SI (Fig. S1 and S3).

### 3.3 Effect of GOx-mediated H_2_O_2_ generation

To assess the influence of enzymatic peroxide generation beyond the linear glucose-to-rate surrogate, we compared the constant-*k* baseline (*k*_deg_ = *k*_0_ + *k*_*G*_*G*) with a mechanistic GOx-mediated variant in which peroxide availability (and thus the effective degradation rate) evolves in time (Section 2.3). A direct comparison is not unique because the GOx pathway introduces a transient build-up of H_2_O_2_ starting from near-zero initial conditions, whereas the constant-*k* model assumes an immediate, time-independent effective stress level.

For a like-for-like evaluation focused on practical read-out comparability, we therefore apply an end-point matching (equivalently, an area-matching in the exponential-integrated rate) in which the GOx-mediated degradation history is rescaled so that the integrated degradation exposure over the observation window matches that of the constant-*k* surrogate at the same glucose level. This procedure guarantees near-identical end-point signals (and thus directly comparable single-time read-outs), while intentionally preserving the distinct transient shape induced by enzymatic kinetics and, in the 1D case, transport effects. Consequently, the remaining discrepancy between trajectories is expected to appear primarily as a transient RMS difference rather than as a systematic end-point bias.

**Fig. 4:**
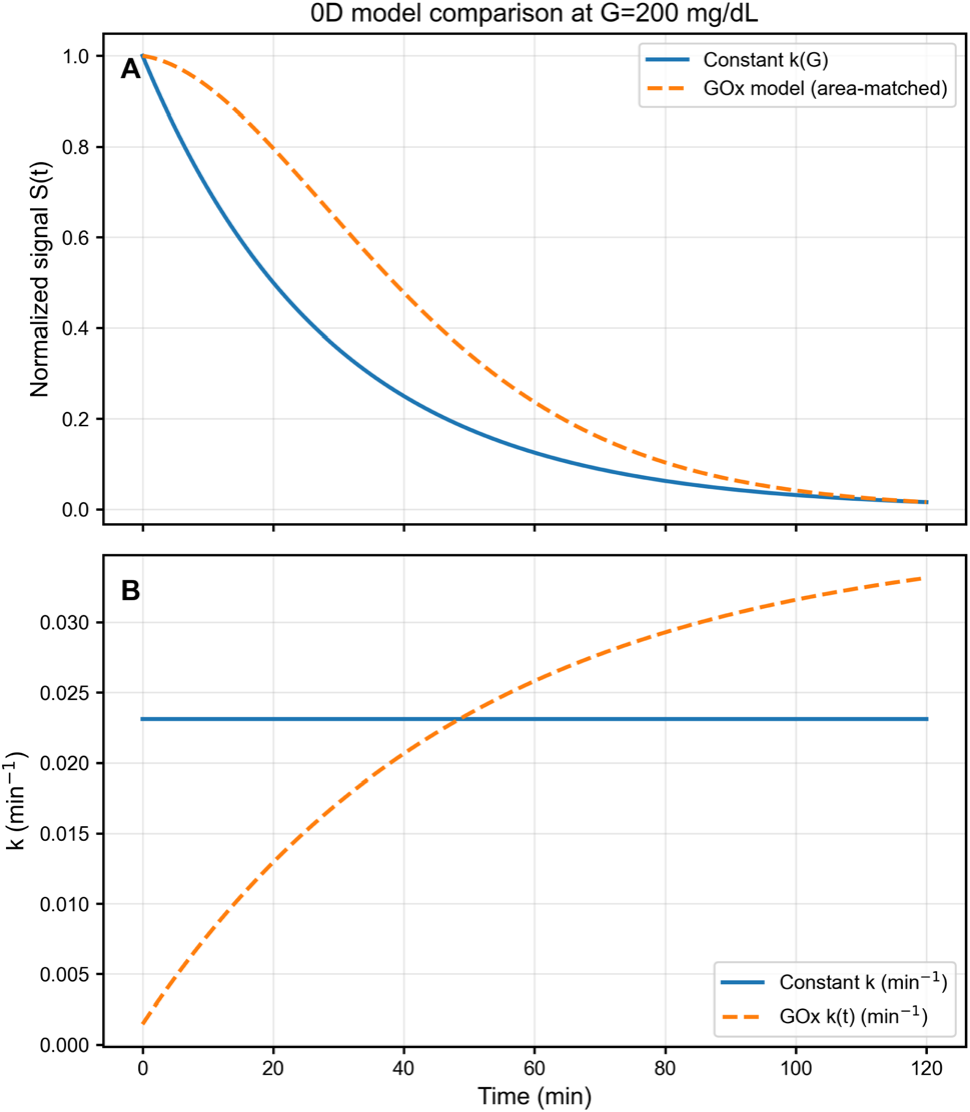
Comparison between the reduced-order constant-*k* degradation model and the GOx-mediated model at *G* = 200 mg dL^−1^ (0–120 min). (A) Signal trajectories: the GOx variant exhibits an early-time lag due to transient H_2_O_2_ build-up while remaining end-point matched over the observation window. (B) Degradation rate profiles: the time-dependent GOx rate *k*(*t*) (dashed red) builds gradually from basal *k*_0_ and is rescaled to match the constant-*k* integrated exposure over the observation window, thereby isolating transient-shape effects from end-point comparability.

Fig. 4 shows results for *G* = 11.1 mM (200 mg dL^−1^) over 0–120 min. Panel (A) compares signal trajectories: the GOx-mediated model exhibits an early-time lag relative to the constant-*k* surrogate, reflecting the finite time required for H_2_O_2_ to accumulate from near-zero initial conditions. Panel (B) displays the corresponding degradation–rate profiles, showing that the GOx-mediated rate starts below the constant-*k* baseline and then increases progressively; under the adopted area-matching procedure, it crosses the constant-*k* level at intermediate times and becomes slightly larger at later times so that the total integrated degradation exposure over the observation window is matched. Importantly, both formulations preserve the same qualitative sensing logic—monotonic signal loss and glucose-dependent kinetic contrast—but differ in their detailed time profile. In this sense, the constant-*k* model provides a convenient reduced-order baseline for design sweeps and optimisation, whereas the GOx model adds biochemical structure when transient dynamics are of interest. A broader comparison between the linearised glucose-to-peroxide mapping and the full Michaelis–Menten production law across representative *K*_M_ values is provided in the SI (Fig. S4).

To assess more directly whether nonlinear Michaelis–Menten saturation materially affects discrimination performance, an additional SI analysis is provided in Fig. S5 of the SI. There, the GOx-mediated model is compared against the linear surrogate across representative *K*_M_ values using the same noise level and practical read-out constraint adopted in the main design study. The results show that saturation can quantitatively increase the minimum classification error, especially for closer glucose pairs, because it compresses the effective peroxide contrast between glucose levels. At the same time, the signal trajectories remain monotonic, and the optimal read-out window stays within a practical tens-of-minutes range over the explored *K*_M_ values.

### 3.4 Discrimination performance and optimal read-out timing

To assess glucose discrimination capability under realistic measurement conditions, we computed the time-dependent signal difference Δ*S*(*t*) = |*S*_1_(*t*) − *S*_2_(*t*)| between representative glucose pairs and the corresponding theoretical classification error assuming Gaussian measurement noise with standard deviation *σ*_*S*_ = 0.02 (2% of normalized scale).

**Fig. 5:**
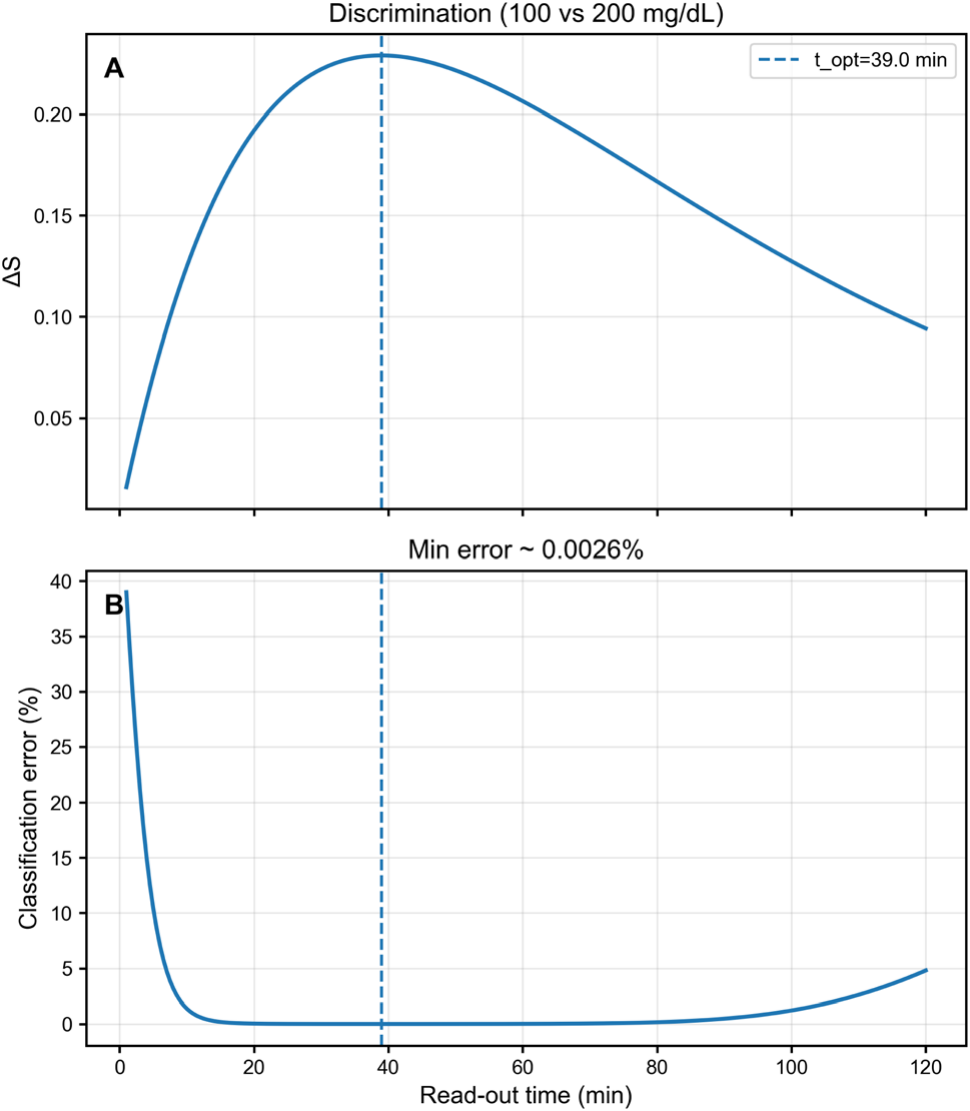
Time-dependent discrimination analysis for glucose pair (100 *vs.* 200 mg dL^−1^). (A) Signal difference Δ*S*(*t*) shows optimal window around *t* ≈ 40 min (vertical dashed line marks *t*_opt_ = 39 min). (B) Theoretical classification error *P*_error_(*t*) reaches minimum of <0.01% at *t*_opt_, demonstrating excellent separability under 2% measurement noise.

Fig. 5 shows the discrimination analysis for the 100 *vs.* 200 mg dL^−1^ pair. Panel (A) displays Δ*S*(*t*), which initially increases as the glucose-dependent degradation contrast accumulates, peaks at intermediate times, and then decreases as both signals approach complete degradation. Panel (B) shows the theoretical classification error *P*_error_(*t*), calculated from the sensitivity index 
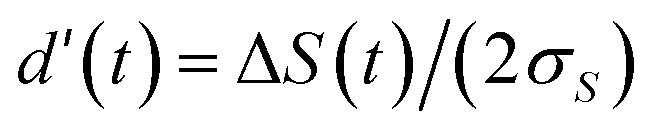
 assuming optimal threshold placement. The minimum error occurs at *t*_opt_ ≈ 39 min, where *P*_error_ < 0.01%, confirming excellent separability at practical read-out times.

This analysis quantifies the trade-off between early read-out (insufficient kinetic contrast) and late read-out (low absolute signal), identifying an optimal window where discrimination is maximized. For the explored parameter regime, optimal read-out times consistently fall in the 30–50 min range across glucose pair combinations, supporting the single-shot measurement strategy proposed in this work.

Assuming independent additive noise with standard deviation *σ*_*S*_ on each signal measurement, the uncertainty of the inter-class separation Δ*S* is 
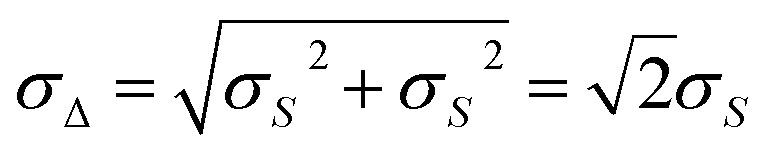
, hence *d*′(*t*) = Δ*S*(*t*)/*σ*_Δ_.

### 3.5 Robustness under measurement noise: Monte Carlo validation

**Fig. 6:**
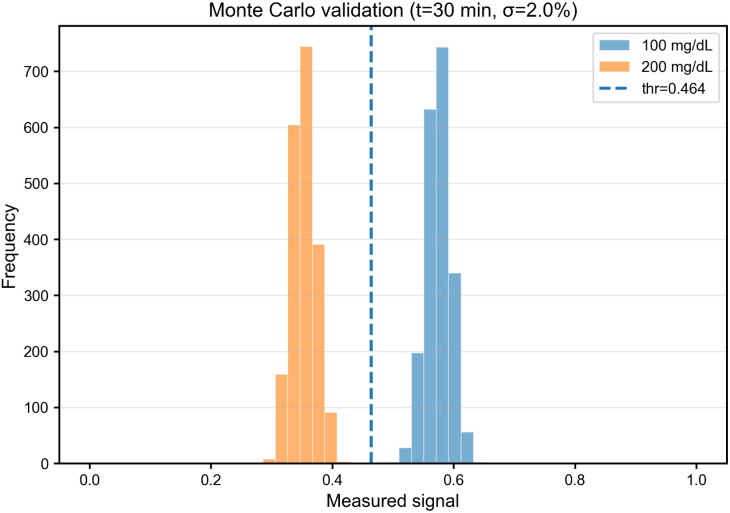
Monte Carlo validation of glucose classification under 2% measurement noise. Histograms show distributions of measured signals *S*_meas_(*t* = 30 min) for 100 and 200 mg dL^−1^ (*N* = 2000 samples per class). The vertical dashed line indicates the optimal threshold. Threshold-based classification achieves 99.0% accuracy despite realistic experimental variability, confirming robust discrimination performance.

To validate the discrimination analysis under realistic experimental variability, we performed Monte Carlo simulations with *N* = 2000 independent realizations per glucose class, each corrupted by additive Gaussian noise (*σ*_*S*_ = 0.02). Fig. 6 shows the resulting distributions of measured signals at *t*_read_ = 30 min for 100 and 200 mg dL^−1^. Despite the measurement variability, the distributions remain well separated. Using a simple threshold classifier positioned at the midpoint between the means (vertical dashed line), we obtain classification accuracy exceeding 99%, consistent with the theoretical prediction from Section 3.4.

This result confirms that the degradation-driven transduction mechanism provides robust glucose discrimination under noise levels representative of low-complexity optical read-out implementations (*e.g.*, LED excitation with photodiode detection or smartphone-based imaging). Higher noise levels (*σ*_*S*_ > 0.05) would require longer observation times or multi-point temporal sampling to maintain comparable accuracy.

### 3.6 Design space optimization

To provide quantitative design guidelines, we performed a grid search over the parameter space (*t*^base^_1/2_, *t*^ref^_1/2_, *β*), evaluating the average classification error across three glucose pairs: (100, 140), (140, 200), and (100, 200) mg dL^−1^. For each configuration, the minimum error within a practical read-out window (*t*_read_ ≤ 60 min) was computed and averaged over pairs.

**Fig. 7:**
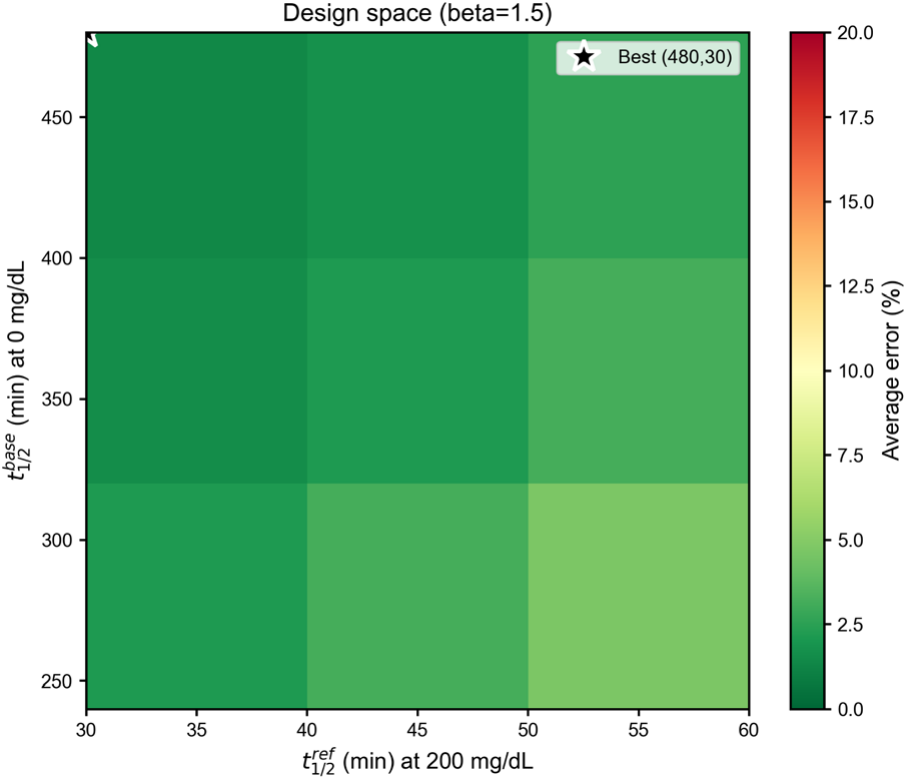
Design space optimization map showing average classification error (in %) as a function of half-life parameters for *β* = 1.5. The optimal configuration (*t*^base^_1/2_ = 480 min, *t*^ref^_1/2_ = 30 min, marked with white star) achieves 1.4% average error. The broad green optimal region demonstrates robustness to parameter variations. Color scale: green = low error (high performance), red = high error.

Fig. 7 shows the resulting design map for *β* = 1.5 (the optimal value identified in the full 3D search). The color scale indicates average classification error in percent, with green regions representing low error (high performance) and red regions high error. The best-performing configuration is (*t*^base^_1/2_, *t*^ref^_1/2_) = (480, 30) min (marked with white star), yielding an average error of 1.4%.

The map reveals several design principles: (i) increasing the half-life contrast *t*^base^_1/2_/*t*^ref^_1/2_ improves discrimination, (ii) excessively fast degradation at high glucose (*t*^ref^_1/2_ < 30 min) limits the usable read-out window, and (iii) a wide plateau of near-optimal configurations exists, indicating that the sensing mechanism does not require fine parameter tuning. This interpretation is consistent with the SI sensitivity analyses: Fig. S1 shows that varying *β* reshapes the temporal response window, with smaller *β* values broadening the response and shifting the optimal read-out to later times, and larger *β* values sharpening early-time decay and compressing the useful window toward earlier times. Over the representative cases explored, *β* ≈ 1.5 lies within a broad low-error regime while keeping the optimal read-out window in a practically convenient range. Fig. S3 further indicates that the practical read-out window remains in the same range under independent moderate perturbations of the nominal kinetic parameters *k*_0_ and *k*_*G*_.

Across the explored grid, optimal read-out times ranged from 34 to 47 min, confirming the 30–50 min window as a robust target for experimental implementation. Table 1 summarizes the per-pair performance metrics for the optimal configuration.

**Table 1:** Discrimination performance for representative glucose pairs at the optimal configuration. Glucose pairs are reported as *G* in mM with the corresponding value in mg dL^−1^ in parentheses (eqn (2))

Pair (mM (mg dL^−1^))	Δ*S*_max_	*t*_opt_ (min)	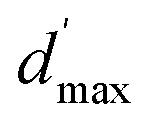	Error (%)	Accuracy (%)
5.55(100) *vs.* 7.77(140)	0.11	47	3.9	2.5	97.5
7.77(140) *vs.* 11.10(200)	0.12	41	4.3	1.6	98.4
5.55(100) *vs.* 11.10(200)	0.23	38	8.1	0.003	99.997

### 3.7 Surface-driven peroxide gradients and depth-dependent degradation

To assess whether finite film thickness and transport limitations can induce spatial heterogeneity, we extended the model to a one-dimensional reaction-diffusion formulation (Section 2.7). Peroxide is generated at the exposed surface *via* GOx kinetics, diffuses into the film with effective coefficient *D*_*H*_ = 10^−18^ m^2^ s^−1^, and is consumed by first-order kinetics. Local material integrity evolves according to the peroxide-dependent rate field *k*(*x*, *t*) = *k*_0_ + *α*_*H*_*H*(*x*, *t*).

**Fig. 8:**
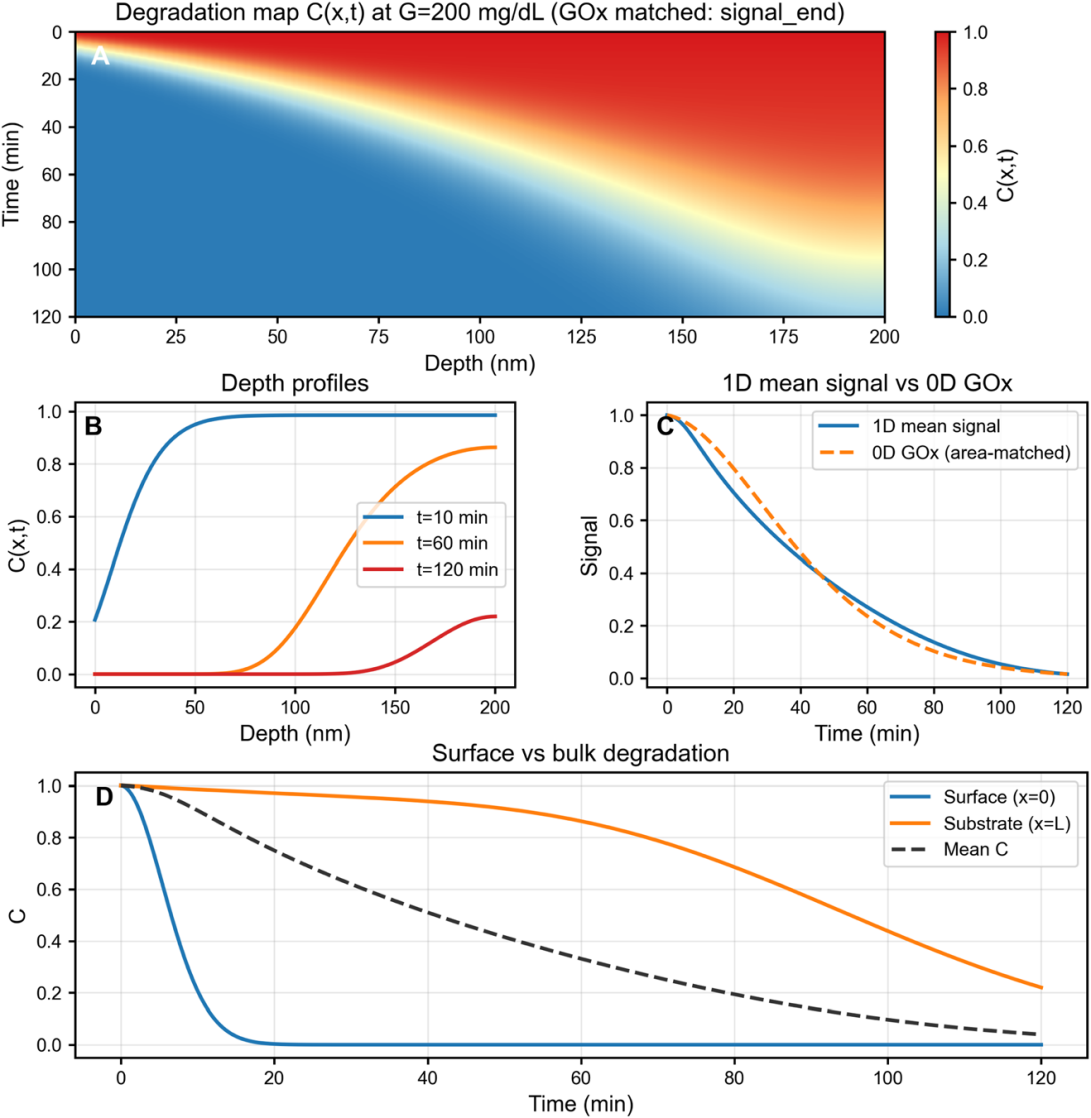
One-dimensional reaction–diffusion analysis revealing surface-driven degradation gradients. (A) Spatiotemporal integrity map *C*(*x*, *t*) (1 = intact, 0 = degraded) showing degradation initiating at the exposed surface (*x* = 0) and progressively penetrating toward the substrate (*x* = *L*). (B) Depth profiles at *t* ≈ 10, 60, 120 min highlighting a moving degradation front and sustained spatial heterogeneity. (C) Thickness-averaged optical readout *S̄*(*t*) = 〈*C*(*x*, *t*)*β*〉*x* compared with the 0D GOx area-matched surrogate. (D) Surface (*x* = 0), substrate (*x* = *L*), and spatially averaged integrity trajectories quantifying heterogeneity while yielding a smooth macroscopic observable. Simulation parameters: *G* = 200 mg dL^−1^, *L* = 200 nm, *D*_*H*_ = 10^−18^ m^2^ s^−1^, *k*_cons_ = 0.02 min^−1^.

Fig. 8 presents a four-panel analysis for *G* = 200 mg dL^−1^ over 0–120 min in a 200 nm film. Panel (A) shows the full degradation map *C*(*x*, *t*), revealing pronounced surface-initiated breakdown driven by elevated peroxide near the exposed interface. The color gradient from red (intact, *C* ≈ 1) to blue (degraded, *C* ≈ 0) visualizes the progressive advance of degradation from the exposed surface into the film. Panel (B) displays depth profiles at representative times (10, 60, 120 min), confirming sustained near-surface gradients due to the competition between peroxide generation, diffusion, and consumption. Already at early times, the exposed region degrades more rapidly than the deeper portion of the film, and this heterogeneity becomes more pronounced as time increases. By later times, the degradation front has penetrated substantially into the film, while the substrate-side region remains comparatively more preserved.

Panel (C) compares the thickness-averaged signal *S̄*(*t*) from the 1D model (solid blue line) with the 0D GOx-matched surrogate (dashed red line); the two curves remain in close agreement, indicating that the macroscopic read-out is adequately captured by the reduced-order formulation despite internal gradients. The small deviation at late times reflects the additional transport resistance in the 1D model, but both predictions remain monotonically decreasing and well-separated across glucose levels.

Panel (D) contrasts surface (*x* = 0), bulk (*x* = *L*), and mean integrity trajectories, illustrating the spatial heterogeneity while confirming that the thickness-averaged response remains smooth and monotonic. The exposed surface degrades most rapidly due to direct peroxide access, whereas the substrate-side region responds more slowly because peroxide penetration is transport-limited.

These results establish two key design implications. First, under the transport regime explored here (slow effective diffusion, first-order consumption), degradation localizes near the surface, consistent with the enzymatic generation occurring at the exposed interface. Second, despite this spatial structure, the thickness-averaged optical signal remains monotonic and well-separated across glucose levels, supporting the use of simple macroscopic read-out for practical implementations. The 0D model is therefore adequate for rapid design screening of read-out windows and kinetic contrast, while the 1D formulation becomes essential when targeting specific film thicknesses, encapsulation strategies, or porous architectures where transport heterogeneity may materially affect the response. A broader sweep over *D*_*H*_–*k*_cons_ space is reported in the SI (Fig. S2), where the transition from strongly surface-localised degradation to the nearly homogeneous limit is mapped explicitly.

### 3.8 SI sensitivity analyses

Additional sensitivity analyses are provided in the SI to assess the robustness of the main design conclusions. Fig. S1 examines sensitivity to the optical exponent *β*, showing that smaller *β* values broaden the temporal response and shift the optimal read-out to later times, whereas larger *β* values sharpen early-time decay and compress the response toward earlier times. Across the representative cases explored, *β* ≈ 1.5 lies within a broad low-error regime while keeping the optimal read-out window in a practically convenient range. Fig. S2 maps transport-induced heterogeneity over *D*_*H*_–*k*_cons_ space, identifying the transition from strongly surface-localised degradation to the nearly homogeneous limit. Fig. S3 quantifies robustness to independent moderate perturbations in the nominal kinetic parameters *k*_0_ and *k*_*G*_, indicating that the practical read-out window remains in the same range across the explored kinetic uncertainty. Fig. S4 compares the linear glucose-to-peroxide mapping against the full Michaelis–Menten production law across representative *K*_M_ values. Fig. S5 further quantifies how Michaelis–Menten saturation affects discrimination performance, showing that saturation can increase classification error—especially for closer glucose pairs—while preserving monotonic signal separation and a practical read-out window over the explored range. Together, these SI analyses indicate that the core qualitative design conclusions remain valid across reasonable variations in optical nonlinearity, transport regime, kinetic uncertainty, and enzymatic saturation, although the quantitative discrimination performance can deteriorate under stronger Michaelis–Menten saturation, especially for closer glucose pairs.

## 4 Discussion

The results of the present model provide design-oriented insight into the feasibility and key trade-offs of degradation-driven glucose sensing. Although the framework is intentionally minimal, the simulated behaviours clarify the conditions under which a perovskite-like layer could act as a time-resolved indicator of glucose concentration through controlled, analyte-dependent signal loss.

### 4.1 Interpretation of the simulated sensor response

Across the explored parameter ranges, the characteristic decay of the optical signal is governed by the effective degradation rate *k*_deg_(*G*) = *k*_0_ + *k*_*G*_*G*, with *k*_*G*_≡*k*_1_*α*. When the glucose-driven contribution is sufficiently large relative to basal degradation, the temporal separation between representative glucose levels becomes apparent at moderate read-out times. In this regime, the degradation profile itself becomes an informative quantitative feature for glucose estimation, consistent with the monotonic single-time read-out responses and the discrimination analyses reported in the Results. From a diagnostic viewpoint, these behaviours translate into well-defined model-derived metrics (Table 1), which formalise how degradation dynamics can be exploited as a sensing feature rather than treated exclusively as a failure mode.

The results also highlight an expected trade-off between sensitivity and usable operating window. Increased susceptibility to peroxide (larger *k*_*G*_) enhances responsiveness but shortens the time interval in which the signal remains informative; conversely, low susceptibility provides stability but reduces separation between glucose levels within practical read-out times. These opposing tendencies define a design space in which the balance between durability and discrimination must be selected according to the intended use case and the available read-out protocol (single-time *vs.* time-resolved).

### 4.2 Scope of the reduced-order approximation

The reduced-order model is best interpreted as a screening-level digital twin that links glucose-driven oxidative stress to a measurable optical response within a controlled operating window. In particular, the prescribed half-lives are design targets rather than fitted material constants, and the linear glucose-to-peroxide mapping is an analytically convenient approximation whose validity depends on the effective enzymatic regime. The SI Michaelis–Menten analysis (Fig. S4) shows that nonlinear saturation changes the detailed transient trajectories and generally shifts the system toward less aggressive degradation than the linearised model, while preserving the monotonic separation principle over the explored range.

The SI discrimination analysis in Fig. S5 makes this point more explicit at the level of performance metrics. In particular, once the GOx-mediated model is matched to the linear surrogate at the reference level, lower effective *K*_M_ values compress the glucose-dependent peroxide contrast and can therefore increase the minimum classification error, especially for closer glucose pairs. However, over the explored range, the signal ordering remains monotonic and the optimal read-out window remains within a practically accessible tens-of-minutes interval.

### 4.3 Quantitative design guidelines from optimization analysis

The parametric optimization (Section 3.6) establishes quantitative targets for experimental realization:

#### 4.3.1 Half-life contrast ratio

A ratio *t*^base^_1/2_/*t*^ref^_1/2_ > *rsim*10 provides adequate glucose-dependent kinetic separation. The optimal configuration (480/30 = 16×) balances discrimination performance with practical read-out times.

#### 4.3.2 Read-out window

Optimal measurement times consistently fall in the 30–50 min range across explored configurations. Earlier read-out sacrifices kinetic contrast; later read-out reduces absolute signal and amplifies relative noise.

#### 4.3.3 Optical nonlinearity

The parameter *β* ≈ 1.5 represents a practical intermediate choice for the representative cases explored: it lies within a broad low-error regime while keeping the optimal read-out window in a convenient 30–50 min range. Larger *β* values shift the response toward earlier times and can compress the usable window, whereas smaller *β* values delay contrast build-up and push the optimal read-out to later times. This trend is consistent with the explicit *β* sensitivity analysis reported in Fig. S1 of the SI.

#### 4.3.4 Noise tolerance

Under additive Gaussian measurement noise of 2% standard deviation, Monte Carlo validation confirms robust discrimination (Fig. 6). Several-percent noise floors are plausible in low-complexity optical read-outs (LED + photodiode or smartphone imaging), and can be mitigated by signal averaging and reference normalization.

#### 4.3.5 Spatial modeling requirements

For thin films in the sub-micron regime and for the transport parameters explored here, the 0D model provides an effective screening tool for read-out windows and kinetic contrast targets. A 1D validation is recommended when thickness, porosity, or barrier/encapsulation layers are expected to significantly alter peroxide penetration and generate strong depth gradients.

These guidelines reduce experimental search space by identifying feasible kinetic regimes and read-out strategies prior to material synthesis and device fabrication.

### 4.4 Bridging model and experiment: calibration protocol

To extract model parameters from experimental measurements and enable predictive use of the digital twin, we propose the following minimal calibration procedure:

#### 4.4.1 Stage 1: basal degradation (*k*_0_)

Measure the optical signal *S*(*t*) under glucose-free conditions (or with inhibited GOx activity) over a time window sufficient to capture ∼1 half-life. Fit an exponential *S*(*t*) = *S*_0_ exp(−*βk*_0_*t*) to extract *k*_0_ and *β* jointly, or measure *β* independently *via* controlled degradation with known *k*. In practice, *β* and *k*_0_ can be partially correlated in noisy datasets; fixing *β* from an independent optical calibration or using multi-time fitting improves identifiability.

#### 4.4.2 Stage 2: glucose-dependent coupling (*k*_1_*α*)

Repeat measurement at a known high glucose level (*e.g.*, 200 mg dL^−1^), yielding *k*_ref_ = *k*_0_ + *k*_1_*α* × *G*_ref_. The coupling follows as *k*_1_*α* = (*k*_ref_ − *k*_0_)/*G*_ref_. Equivalently, this step identifies the effective glucose-to-degradation coupling *k*_*G*_, which is the experimentally observable lumped quantity in the reduced-order model.

#### 4.4.3 Stage 3: validation and refinement

Use the calibrated (*k*_0_, *k*_1_*α*, *β*) to predict *S*(*t*; *G*) at intermediate glucose levels. Validate with sparse measurements (*e.g.*, 2–3 additional concentrations at a single read-out time). If significant deviations occur, consider refining with the GOx-mediated formulation or adding a quadratic term in *G* to capture enzymatic saturation.

This three-stage protocol requires only 2–3 full temporal measurements plus sparse validation points, enabling rapid model calibration from limited experimental data.

### 4.5 Design implications of transport-induced gradients

The 1D results indicate that peroxide-driven degradation may localise near the exposed interface when transport is restricted and peroxide is consumed within the matrix. Experimentally, this implies that film thickness, surface functionalisation, and any barrier/encapsulation layers act as key design knobs that can reshape the temporal response. Even when gradients exist, the thickness-averaged signal *S̄*(*t*) remains a practical macroscopic read-out for low-complexity optical measurements; however, spatial localisation suggests that surface chemistry and microstructure may control repeatability and should be considered in prototype design.

The broader transport sweep reported in Fig. S2 of the SI further shows that the nominal configuration used in the main text lies within a heterogeneous regime where surface-to-bulk contrasts are non-negligible, whereas higher *D*_*H*_ and/or lower *k*_cons_ progressively drive the system toward the spatially homogeneous limit. This supports the use of the 1D formulation as a validation tool when transport resistance is expected to matter, while also clarifying when the reduced-order 0D approximation remains adequate.

### 4.6 Model limitations and opportunities for refinement

Several simplifications underlie the present framework: (i) quasi-steady and lumped enzymatic behaviour (through the effective mapping embedded in *α*), (ii) reduced-order degradation kinetics (pseudo–first-order in the intact fraction), and (iii) a compact phenomenological optical response *S*(*t*) = *S*_0_*C*(*t*)^*β*^. These assumptions are appropriate for a first-order, design-oriented feasibility analysis, but they do not capture the full chemical and environmental complexity of real operating conditions. In this sense, the linear glucose-to-peroxide mapping should be viewed as a reduced-order approximation that prioritises analytical transparency; when enzyme saturation, oxygen limitation, or deactivation become important, the full GOx-mediated transient model becomes the more appropriate description.

In particular, the 0D model neglects spatial transport and local gradients of reactive species. While a 1D reaction–diffusion extension was evaluated in the Results, further refinement could incorporate coupled reaction–diffusion with explicit peroxide generation/consumption, humidity- and temperature-dependent rate constants, and multi-step degradation pathways producing intermediate species with distinct optical signatures. Such additions would improve material- and geometry-specific predictiveness, at the expense of additional parameters and potential identifiability challenges. The current formulation therefore prioritises analytical clarity and interpretability, with the goal of identifying feasible kinetic regimes and design trade-offs rather than providing post hoc fitting to specific experimental datasets.

### 4.7 Broader implications

More broadly, the concept explored here aligns with the general paradigm of *function-by-degradation*, in which controlled instability is intentionally harnessed as a design feature. In principle, degradation-triggered sensing could extend beyond glucose to other biochemical targets whose reactivity modifies material stability. In that sense, the present work contributes to a broader shift in which material fragility can be repurposed as functional sensitivity, provided the trigger pathway is sufficiently controlled and interpretable.

Although the model is formulated in a material-agnostic manner, the candidate families discussed in this work provide experimentally plausible anchors for the explored kinetic regimes. Several lead-free perovskite and perovskite-inspired compounds represent realistic candidates for future experimental exploration. Double perovskites such as Cs_2_AgBiBr_6_ and bismuth-based halides (A_3_Bi_2_Br_9_, A_3_Bi_2_I_9_ with A = Cs, MA, FA) combine suitable optical activity with known sensitivity toward moisture and oxidising agents, including peroxide-related stress. Tin-based hybrid perovskites (*e.g.*, FASnI_3_, MASnI_3_) also exhibit rapid oxidative degradation and could support fast responses, but their handling constraints and environmental sensitivity are non-trivial. Lead-based perovskites are excluded here due to application-driven safety constraints for skin-interfaced or transdermal formats.

### 4.8 From theoretical framework to experimental validation

Although the present work is intentionally theoretical, the proposed degradation-driven sensing concept is compatible with straightforward proof-of-concept experiments using established fabrication and enzymatic immobilisation approaches. Lead-free perovskite and perovskite-inspired materials can be processed into thin films using low-temperature solution routes (*e.g.*, spin coating or drop casting) on rigid or flexible substrates. GOx can be immobilised at the surface or within a permeable polymer matrix using crosslinking or entrapment strategies commonly employed in enzymatic biosensors.

Optical read-out can be implemented as an intensity-based measurement (*e.g.*, photoluminescence quenching or absorbance changes) without requiring integrated electronics, provided that illumination, collection geometry, and background variations are controlled. Initial validation could focus on *in vitro* assays in which glucose concentration and relevant environmental factors (*e.g.*, humidity) are independently varied to test the predicted kinetic contrast and candidate read-out windows identified by the model.

Importantly, while the present study does not claim device-level performance, it does provide quantitative relationships that can help narrow the experimental search space by linking kinetic regimes, read-out timing, and the required contrast between basal and glucose-driven degradation. In this sense, the model offers a practical starting point for selecting material stability targets, designing assay timing, and prioritising parameter ranges before more detailed experimental optimisation.

### 4.9 Shelf-state protection and deployment considerations

Because the present concept exploits degradation only during the measurement window, pre-use shelf stability should be protected rather than exposed to the same stressors used for sensing. Practical low-complexity strategies include dry packaging, desiccant-assisted storage, barrier laminates, or sealed single-use cartridges that isolate the sensing layer until deployment. In this way, instability is operationally activated during use rather than during storage, which is particularly relevant for disposable patch-like or wound-facing formats.

### 4.10 Degradation-driven biosensors as material-level digital twins

Beyond its immediate application to glucose sensing, the modelling framework developed in this work can be interpreted as a material-level digital twin of a degradation-driven biosensor. Here, the digital twin is not a high-fidelity replica of a complex device, but a minimal, dynamically coupled representation that links biochemical input, material state, and observable output.

The physical entity is the perovskite-like sensing layer functionalised with glucose oxidase, while the digital counterpart is a reduced-order dynamical model describing peroxide-linked degradation kinetics and optical signal decay. The latent degradation state *C*(*t*) acts as an internal variable governing the measured signal, providing a structured mapping between hidden material evolution and accessible read-outs. By enabling parameter sweeps, sensitivity analysis, and identification of candidate operating windows, this digital-twin perspective illustrates how reduced-order models can serve as design and optimisation tools for disposable sensors where controlled degradation is an essential functional element rather than an undesired ageing process.

## 5 Conclusions

This work develops a design-oriented degradation-driven modelling framework for glucose sensing in which controlled chemical instability of lead-free perovskite-like materials is treated as the transduction mechanism. By coupling Michaelis–Menten GOx kinetics to peroxide-accelerated pseudo-first-order degradation and a compact optical response model, we obtain closed-form signal trajectories that capture the central sensing logic: higher glucose increases the effective degradation rate and produces systematically faster optical signal decay. Within the tested parameter regimes, the model yields monotonic signal-concentration relations at fixed read-out times, supporting the feasibility of discrimination between representative glycaemic ranges using simple intensity-based optical read-out.

A complementary one-dimensional reaction-diffusion formulation was used to probe the impact of spatial heterogeneity across thin films. The simulations indicate that depth-dependent degradation can generate gradients, yet the thickness-averaged signal remains a useful summary observable for sensor-level read-out and design exploration. Monte Carlo analyses with additive measurement noise suggest that class separation can remain robust under the assumed noise levels (2% standard deviation, >*rbin*99% accuracy for 100 *vs.* 200 mg dL^−1^).

Parameter space optimization identifies an optimal configuration (basal half-life 480 min, accelerated half-life 30 min at 200 mg dL^−1^, optical exponent *β* = 1.5) yielding 1.4% average classification error across three clinically relevant glucose pairs, with optimal read-out times in the 30–50 min range. Design guidelines derived from this analysis establish quantitative targets: half-life contrast ratio >10×, optical nonlinearity *β* ≈ 1.5, noise tolerance up to 5%, and the practical adequacy of 0D screening for thin films under the transport conditions explored here, with the 1D model serving to validate thickness choices and assess encapsulation-dependent peroxide penetration. The half-life values explored here should therefore be interpreted as experimentally informed design targets for material down-selection and calibration, rather than as validated kinetic constants of a single already-demonstrated glucose-responsive platform.

This supports a practical workflow in which the 0D model is used for rapid optimisation of read-out timing and kinetic contrast, while the 1D transport model is invoked to validate thickness choices and assess the impact of encapsulation-dependent peroxide penetration.

Overall, the results establish a quantitative baseline for exploring disposable biosensing concepts based on lead-free double perovskites and bismuth halides, and they define actionable targets for future proof-of-concept validation *via* thin-film fabrication, GOx immobilisation, and simple optical measurements. More generally, the proposed degradation-driven digital-twin perspective is transferable to other sensing problems in which chemical or biochemical stimuli modulate material stability, enabling reduced-order, data-informed design of time-resolved diagnostic read-outs.

Quantitatively, the framework predicts classification accuracy >*rbin*99% under 2% measurement noise, with Monte Carlo simulations indicating robust performance across 2000 independent realizations per glucose class. The computational infrastructure and design guidelines established here provide a quantitative foundation for experimental down-selection and prototype validation, bridging material-level digital twin concepts with practical biosensing applications where instability is intentionally harnessed as a functional asset.

## Author contributions

E. V. F.: conceptualisation, methodology, software, investigation, formal analysis, visualisation, writing – original draft. S. M. L.: conceptualisation, methodology, supervision, writing – review & editing.

## Conflicts of interest

There are no conflicts to declare.

## Supplementary Material

RA-016-D6RA01076H-s001

RA-016-D6RA01076H-s002

RA-016-D6RA01076H-s003

RA-016-D6RA01076H-s004

RA-016-D6RA01076H-s005

RA-016-D6RA01076H-s006

RA-016-D6RA01076H-s007

## Data Availability

All equations, parameter values, and simulation settings needed to reproduce the results are provided in the manuscript and the supplementary information (SI). The code used to generate the model results and figures reported in this paper is available at Zenodo: https://doi.org/10.5281/zenodo.18444878 No new experimental data were generated; all results are reproducible from the deposited code.^50–61^ Supplementary information: practical optical read-out considerations, candidate-material context, summary parameter tables, and supplementary sensitivity analyses on optical nonlinearity, transport regime, kinetic uncertainty, and Michaelis–Menten saturation. See DOI: https://doi.org/10.1039/d6ra01076h.
