# Supplementary figures and images for: Degradation-as-signal: a digital-twin framework for disposable optical glucose sensing with lead-free perovskite-inspired films
Source: RSC Adv. 2026 Apr 22;16(23):20908–22. doi: 10.1039/d6ra01076h (PMC13101435; doi:10.1039/d6ra01076h)

**Figure S1 - Sensitivity to optical exponent  $\beta$**

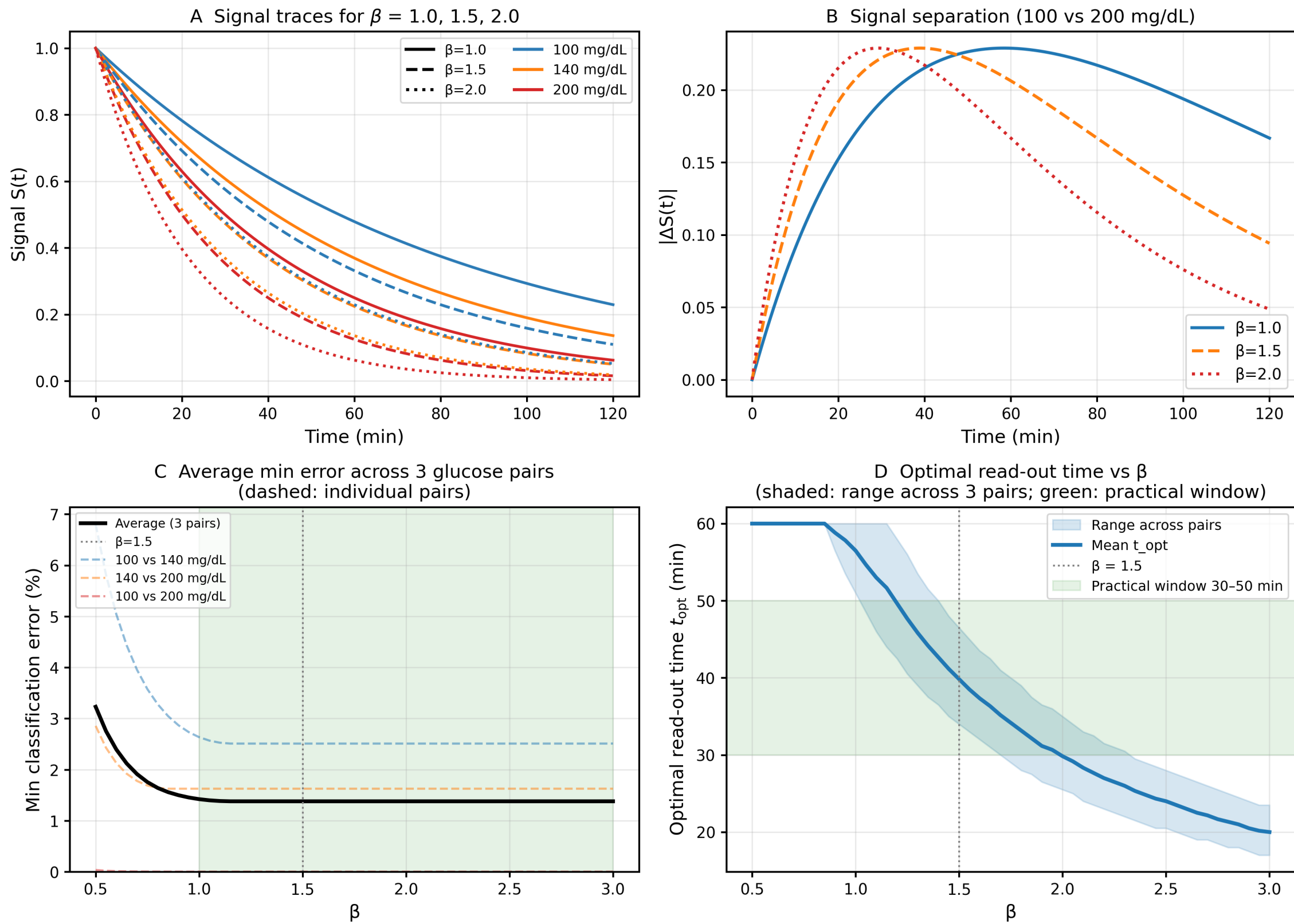

Supplement: RA-016-D6RA01076H-s002 [file RA-016-D6RA01076H-s002.pdf]

**Figure S2 – Transport-regime parameter sweep:  $D_{\text{eff}} \times k_{\text{cons}}$  (1D GOx model, unscaled)**

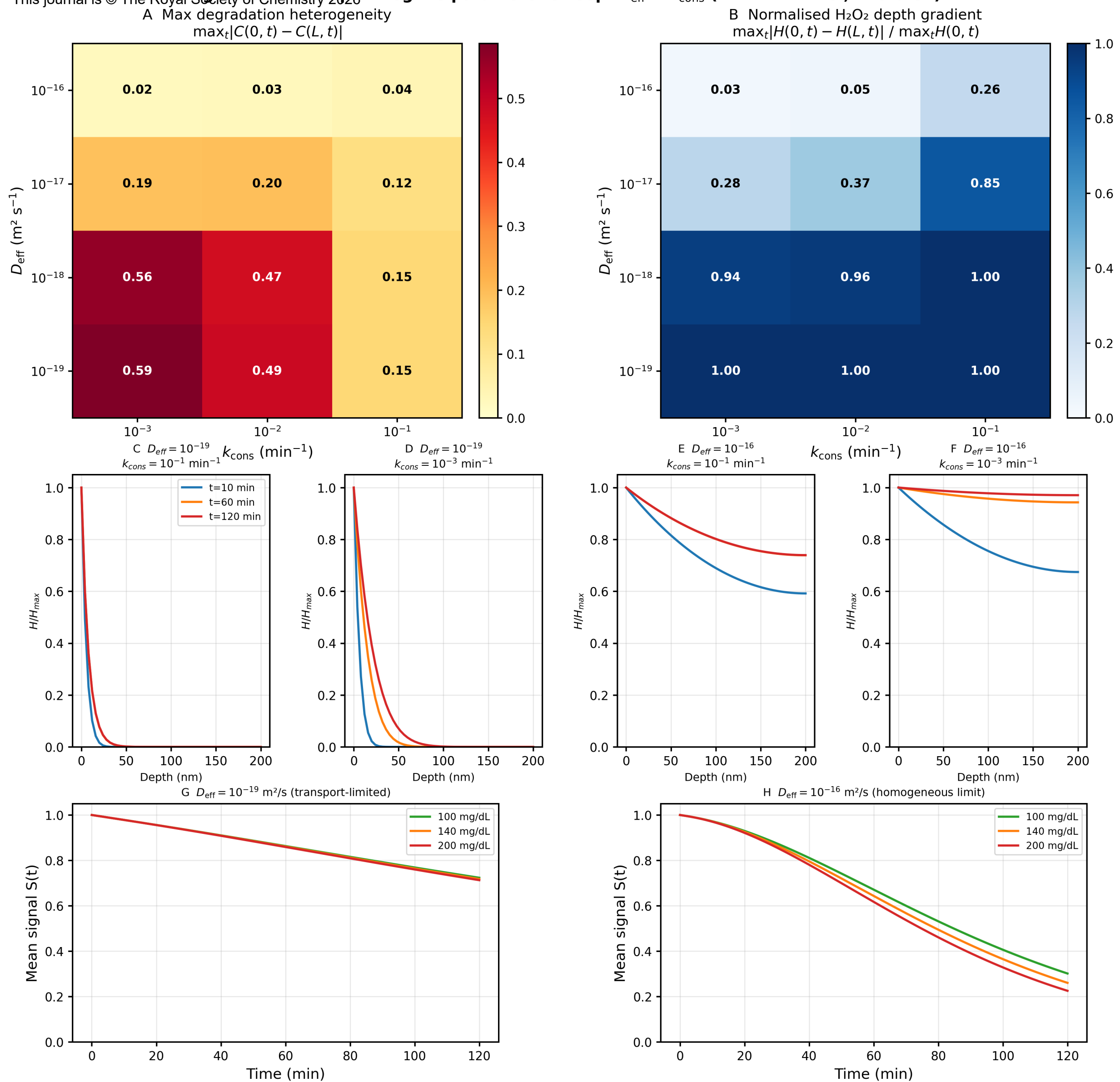

Supplement: RA-016-D6RA01076H-s003 [file RA-016-D6RA01076H-s003.pdf]

# Figure S3 – Kinetic robustness: $\pm 30\%$ perturbation on $k_0$ and $k_G$

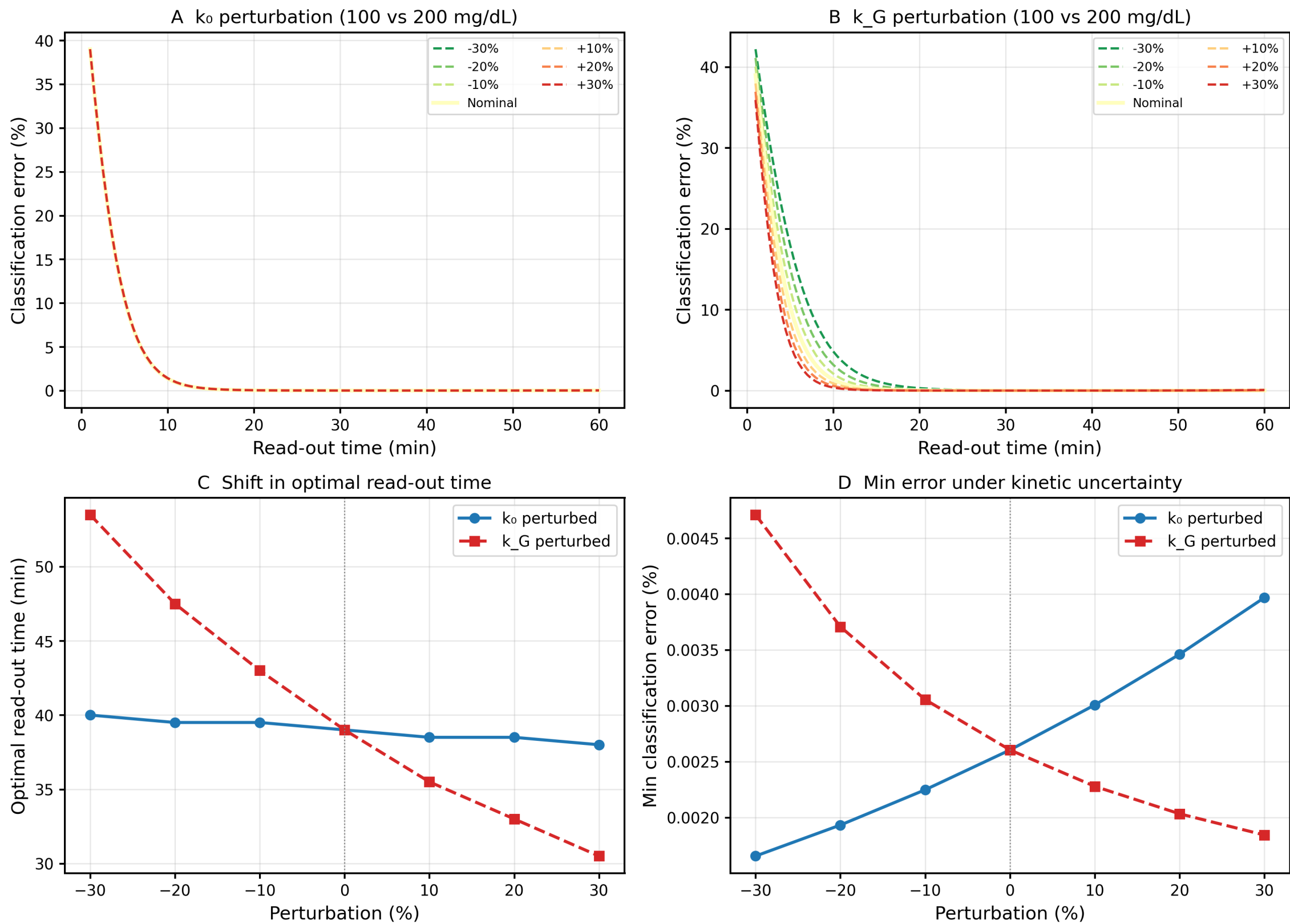

Supplement: RA-016-D6RA01076H-s004 [file RA-016-D6RA01076H-s004.pdf]
