# Supplementary material for: Degradation-as-signal: a digital-twin framework for disposable optical glucose sensing with lead-free perovskite-inspired films
Source: RSC Adv. 2026 Apr 22;16(23):20908–22. doi: 10.1039/d6ra01076h (PMC13101435; doi:10.1039/d6ra01076h)

This journal is © The Royal Society of Chemistry 2026

Figure S4 – Linearised vs full Michaelis–Menten GOx model
Linearisation is a conservative upper bound: $H_{lin} \geq H^{MM}$

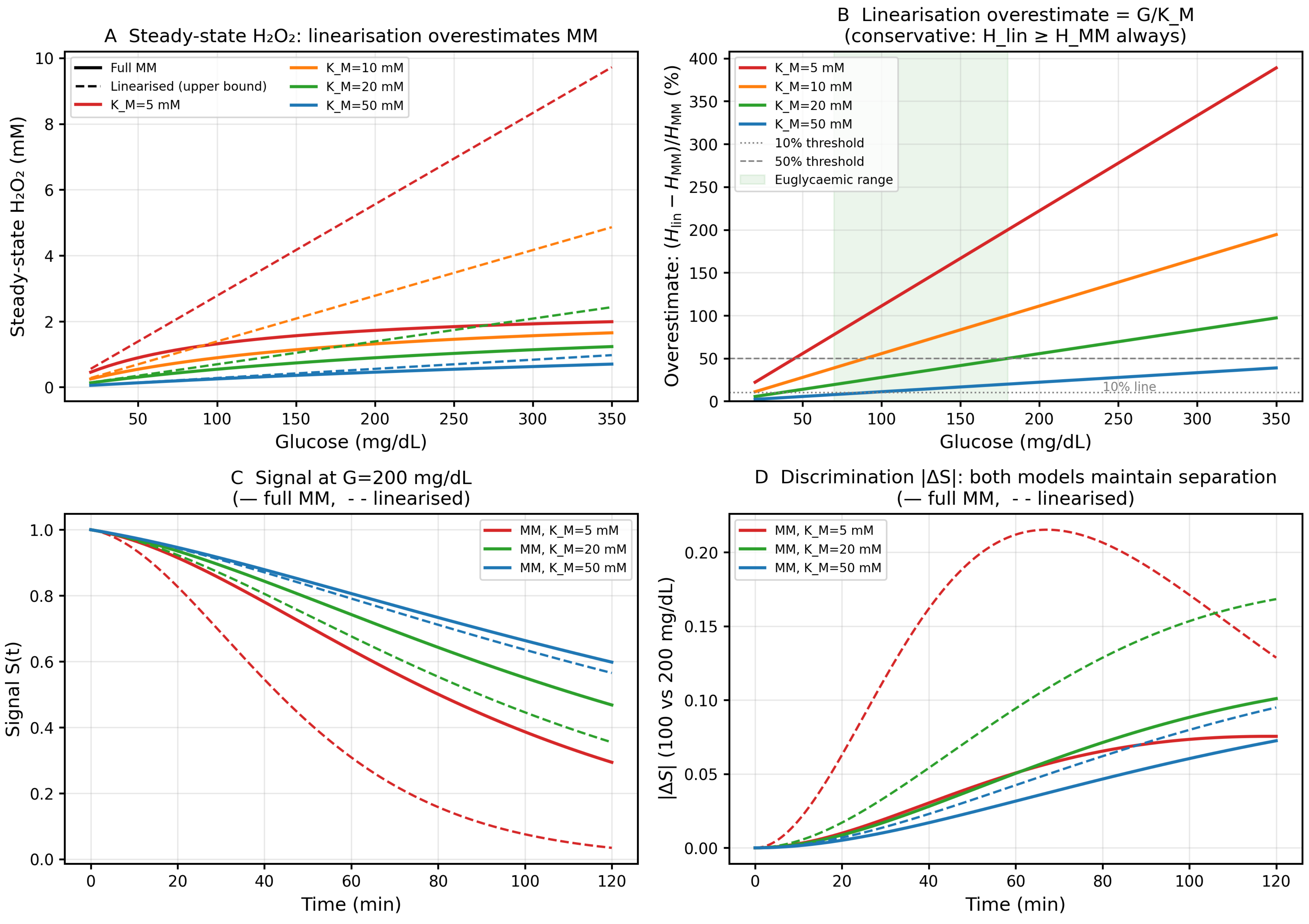

Supplement: RA-016-D6RA01076H-s005 [file RA-016-D6RA01076H-s005.pdf]
