# Supplementary material for: Degradation-as-signal: a digital-twin framework for disposable optical glucose sensing with lead-free perovskite-inspired films
Source: RSC Adv. 2026 Apr 22;16(23):20908–22. doi: 10.1039/d6ra01076h (PMC13101435; doi:10.1039/d6ra01076h)

This journal is © The Royal Society of Chemistry 2026

Figure S5 – Impact of Michaelis–Menten enzymatic saturation on discrimination performance (area-matched at $G_{ref} = 200$ mg/dL; $\sigma_S = 2\%$; $t_{read} \leq 60$ min)

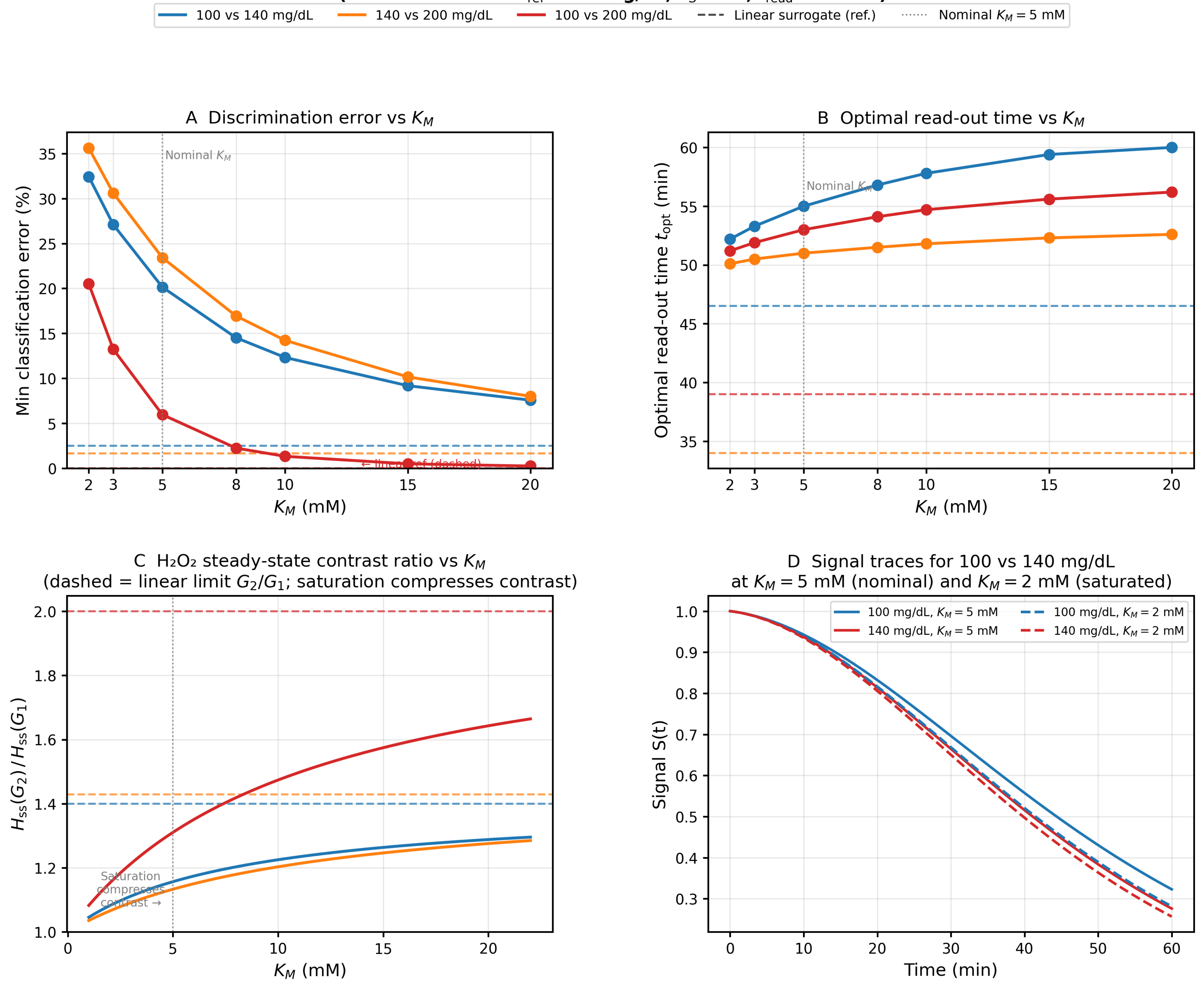

Supplement: RA-016-D6RA01076H-s006 [file RA-016-D6RA01076H-s006.pdf]
