# Supplementary material for: Degradation-as-signal: a digital-twin framework for disposable optical glucose sensing with lead-free perovskite-inspired films
Source: RSC Adv. 2026 Apr 22;16(23):20908–22. doi: 10.1039/d6ra01076h (PMC13101435; doi:10.1039/d6ra01076h)
Supplement: RA-016-D6RA01076H-s007 [file RA-016-D6RA01076H-s007.pdf]

Supplementary Information (SI) for RSC Advances.
This journal is © The Royal Society of Chemistry 2026

Electronic Supplementary Information (ESI)

Degradation-as-Signal: A Digital-Twin Framework for Disposable Optical Glucose Sensing with Lead-Free Perovskite-Inspired Films

Erika Vega-Fleitas and Santiago Moll-López

S1 Practical optical read-out implementation (informative)

S1.1 Candidate observables.

The model variable $S(t)$ represents a normalised optical read-out that decreases as the perovskite-like layer degrades. Experimentally, $S(t)$ may correspond to: (i) integrated photoluminescence (PL) intensity under LED excitation, (ii) transmittance $T(\lambda, t)$ or absorbance $A(\lambda, t)$ at one or two selected wavelengths near the absorption edge, or (iii) diffuse reflectance (or colourimetric indices) extracted from camera RGB channels under controlled illumination.

S1.2 Minimal instrumentation.

A low-complexity implementation can be achieved with a fixed-geometry clip-on module comprising an LED source (excitation or probe), optional optical filtering (for PL), and either a photodiode or a smartphone camera. For PL, a long-pass filter suppresses the pump; for absorbance/reflectance, one wavelength can be chosen near the absorption edge (signal channel) and a second wavelength far from the edge (reference channel), enabling ratiometric compensation for illumination drift and alignment.

S1.3 Normalisation and internal controls.

To reduce dependence on absolute intensity and collection geometry, measurements are reported as $S(t)/S(0)$ or as a ratiometric signal $S_1(t)/S_2(t)$. In addition, a co-fabricated reference region without GOx (or with inhibited activity) can serve as an internal control to estimate basal degradation (k_0) separately from peroxide-driven contributions, improving robustness under variable humidity and temperature.

S2 Candidate material families and supporting literature

S2.1 Material selection rationale

Although the formulation of the model is material-agnostic, identifying realistic candidate compounds is essential for ensuring that the simulated kinetic regimes correspond to experimentally plausible behaviour. Because the envisioned applications include skin-interfaced patches and wound-monitoring devices, biocompatibility and the absence of toxic elements are fundamental constraints. For this reason, conventional lead-based perovskites (e.g., $MAPbI_3$, $FAPbI_3$, $CsPbBr_3$) are excluded despite their favourable optoelectronic properties.

A first family of suitable materials are the *lead-free double perovskites*, particularly $Cs_2AgBiBr_6$. This compound displays strong optical absorption, moderate photoluminescence, and clear environmental sensitivity under moisture and photo-assisted oxidative stress, which is consistent

with a degradation-driven transduction concept. The formation of AgBr- and BiO_x-rich products under oxidative conditions aligns naturally with the sensing principle proposed in the main text, whereby the rate of degradation encodes glucose concentration. In addition, $Cs_2AgBiBr_6$ has been successfully processed into thick-film and composite architectures suitable for optical devices. In particular, composite $CsPbBr_3/Cs_2AgBiBr_6$ thick films have demonstrated stable photodetector performance in the visible range.[1]

A second group of candidates includes *bismuth-based halides* such as $A_3Bi_2Br_9$ and $A_3Bi_2I_9$ (A = Cs, MA, FA). These perovskite-inspired materials exhibit high sensitivity to humidity and oxidants, a lead-free composition and reported environmental sensitivity, and compatibility with low-cost solution processing. Their ease of fabrication and rapid environmental response make them promising platforms for disposable sensors.

Tin-based hybrid perovskites, such as $MASnI_3$ and $FASnI_3$, constitute a third class of materials whose rapid oxidative degradation could support fast sensor responses. However, their intrinsic instability and air sensitivity require careful handling. Still, when controlled degradation is desired—as in the present concept—such reactivity may become a functional advantage.

The kinetic ranges explored in this study should therefore be interpreted as screening-level design targets spanning plausible slow-versus-accelerated degradation regimes across these lead-free material families, rather than as literature-fitted constants for a single validated glucose-responsive composition.

More generally, ligand/passivation strategies can dramatically improve perovskite nanocrystal stability even in highly polar media; while Pb-based examples exist, the underlying design logic (surface chemistry control) is transferable to lead-free analogues.[2]

S2.2 $Cs_2AgBiBr_6$ literature-grounded plausibility anchors for the simulations

This study highlights $Cs_2AgBiBr_6$ as a representative lead-free double perovskite for which extensive structural and optical data are available[3–6]. The literature values summarised below provide plausibility anchors (e.g., bandgap range, typical film thicknesses, and qualitative environmental sensitivity) for the kinetic regimes explored in the main text. The core simulations in the manuscript are parameterised via prescribed half-lives to enable material-agnostic design sweeps rather than material-specific fitting.

It is important to distinguish qualitative literature anchoring from material-specific kinetic fitting. The studies cited in this section provide plausibility anchors for optical activity, film geometry, and environmental sensitivity, but they do not report directly measured glucose-coupled or H_2O_2-coupled degradation half-lives for a lead-free perovskite thin-film biosensing architecture of the type considered here. Accordingly, the nominal half-life values adopted in the main text are not presented as literature-fitted constants for $Cs_2AgBiBr_6$, but as screening-level design targets used to explore whether a practically useful discrimination window could exist.

S2.2.1 Bandgap and optical transitions.

Pristine $Cs_2AgBiBr_6$ exhibits an indirect bandgap of approximately 2.18 eV, with hydrogenation treatments reducing the bandgap into the 1.7–1.9 eV range (Ref.[6]). Direct optical transitions near 2.1–2.2 eV have been observed in porous and microcrystalline films (Refs.[5,7]). These values place $Cs_2AgBiBr_6$ in the visible region, enabling optical readout of degradation.

S2.2.2 Film morphology and typical thickness.

Thin films used in optoelectronic devices typically exhibit thicknesses of 100–200 nm.[6] Porous architectures formed by stacked nanoplatelets (thickness ∼150 nm, lateral dimensions 10–50 μm) have also been reported.[5] These length scales motivate the use of one-dimensional geometries with total thicknesses of 100–500 nm in diffusion and degradation models.

S2.2.3 Charge transport and carrier dynamics.

Mobilities for $Cs_2AgBiBr_6$ fall in the 1–10 $cm^2V^{-1}s^{-1}$ range, with carrier lifetimes between 18 and 42 ns.[6] Although these values do not directly enter the chemical degradation kinetics, they determine the intrinsic radiative response prior to breakdown.

S2.2.4 Diffusion of small reactive species.

Hydrogen incorporation studies indicate an effective diffusion coefficient of $D \sim 10^{-14}$–10^{-13} cm^2s^{-1}.[6] Although hydrogen is not the target species in the present sensing concept, these values provide order-of-magnitude references for transport of small oxidising or aqueous species.

S2.2.5 Environmental stability and degradation behaviour.

$Cs_2AgBiBr_6$ shows high stability under inert atmosphere and moderate illumination, but degrades rapidly under conditions such as 85°C and 85% RH.[4,6] These observations support the use of reduced-order first-order degradation kinetics as a screening description of slow versus accelerated degradation regimes. However, they should not be interpreted as providing directly measured glucose-coupled or H_2O_2-coupled half-lives in the minute-scale range used in the main text.

S2.3 $A_3Bi_2Br_9$ (A = Cs, MA, FA): supporting literature context

Bismuth-based halide perovskite derivatives of the $A_3Bi_2Br_9$ family (A = Cs^+, MA^+, FA^+) have attracted increasing interest as lead-free platforms for optoelectronic sensing.[8] Their low-dimensional, vacancy-ordered motifs enable relatively wide band gaps in the blue/near-UV range, while maintaining processability and defect-tolerant response in selected configurations.[8] In the main text, bromide $A_3Bi_2Br_9$ compositions are highlighted because they show (i) stimulus-dependent band-edge shifts (temperature/pressure),[9,10] (ii) device-level operation in the violet/blue spectral region,[11–13] and (iii) representative examples of stable operation in radiation detection.[14]

S2.3.1 Dimensionality and stimulus-responsive optical behaviour.

Compounds such as $Cs_3Bi_2Br_9$, $MA_3Bi_2Br_9$, and mixed-halide systems such as $MA_3Bi_2Br_6Cl_3$ naturally adopt 0D or 2D vacancy-ordered architectures, where isolated or layered $BiBr_6$ octahedra confine carriers and suppress long-range ion migration. This structural confinement is advantageous for applications where controlled chemical or photochemical degradation is desired, including the degradation-driven mechanism explored in the main text.

S2.3.2 Thermochromism as an optical transduction channel.

$Cs_3Bi_2Br_9$ and $MA_3Bi_2Br_9$ exhibit reversible temperature-driven optical changes.[9] Reported behaviour includes a band-gap reduction on heating (e.g., a decrease of ∼0.13–0.14 eV between room temperature and 120 °C), accompanied by a visible color change at higher temperatures.[9] This provides a calibrated mapping between an external stimulus and optical observables (absorption edge / perceived color).

S2.3.3 Pressure-driven band-structure modulation and phase sensitivity.

High-pressure studies on $MA_3Bi_2Br_9$ report band-gap narrowing up to several GPa, followed by a blue shift across a pressure-induced structural transition (reported from trigonal to monoclinic symmetry in the ∼4.6–5.5 GPa range).[10] This evidences a strong dependence of optical response on lattice distortion and octahedral connectivity.

S2.3.4 Optoelectronic tunability and violet/UV emission.

$MA_3Bi_2Br_9$ and $MA_3Bi_2Br_6Cl_3$ quantum dots exhibit emission tunable from 379–400 nm, demonstrating bandgap engineering via halide substitution and crystallisation kinetics.[11]

S2.3.5 Photodetection demonstrations in the violet/blue range.

Narrowband blue photodetection has been demonstrated using $MA_3Bi_2Br_9$ architectures exploiting charge-collection narrowing, including reported responsivities around 0.1 mA W^{-1} at 470 nm and spectral widths as low as 24 nm.[12] Dual-band photodetection has been reported via air-induced formation of $Cs_3Bi_2Br_9$/Cs_3BiBr_6 bulk heterojunctions, with two distinct response bands (near-UV and blue) and microsecond-scale response times.[13]

S2.3.6 Electronic transport, defect tolerance, and radiation detection context.

Single-crystal $Cs_3Bi_2Br_9$ has been reported with extremely low dark currents, high resistivity, and excellent X-ray attenuation.[14] Radiation detection using $Cs_3Bi_2Br_9$ single crystals has also been reported with low detection limits and suppressed ionic-migration-related instabilities.[14] Thin-film studies further report ion-migration-mediated resistive switching in sub-100 nm $Cs_3Bi_2Br_9$ films.[15]

S2.3.7 Relevance for degradation-driven sensing and digital-twin calibration.

Collectively, the above studies position $A_3Bi_2Br_9$ (especially $Cs_3Bi_2Br_9$ and $MA_3Bi_2Br_9$) as a practical lead-free testbed where stimulus-induced or degradation-induced structural changes can be mapped into optical/electrical observables.[9–14] This is compatible with a degradation-driven digital-twin framework in which physically meaningful state variables (e.g., k_{deg} and $C(t)$) are linked to an experimentally accessible output $S(t)$ through compact parameters (k_0, $k_1\alpha$ and β). In practice, sparse measurements at one or a few read-out times can be used to calibrate these parameters, after which the digital twin can predict signal trajectories across glucose levels and operating windows.

S2.3.8 Related evidence from lead-free double perovskites.

Reproducible ion migration and defect reconfiguration processes are also consistent with demonstrations of stable bipolar resistive switching in lead-free $Cs_2AgBiBr_6$ memristive devices.[16]

S2.4 Summary of model parameters

The tables below summarize the representative model parameters used throughout the reduced-order and transport-aware analyses, together with their roles in calibration or design-space exploration.

Table S1: Summary of model parameters, representative values, units, and modelling roles used in the reduced-order and transport-aware formulations.

Parameter	Value	Units	Role
Film thickness L	200	nm	fixed
Optical exponent β	1.5	–	design variable
Basal half-life $t_{1/2}^{(0)}$	480	min	design target
Reference half-life $t_{1/2}^{(\mathrm{ref})}$	30	min	design target
Reference glucose G_{ref}	200	$\mathrm{mg\,dL^{-1}}$	fixed (design reference)
K_M	5.0	mM	representative
$V_{\max}$	0.05	$\mathrm{mM\,min^{-1}}$	representative
k_{cons}	0.02	$\mathrm{min^{-1}}$	representative
D_H	10^{-18}	$\mathrm{m^2\,s^{-1}}$	representative
Noise standard deviation σ_S	0.02	–	fixed
1D grid points N_x	101	–	numerical
0D time step Δt_{0D}	0.1	min	numerical

Derived quantities such as k_0 and k_G are obtained from the prescribed half-lives in the reduced-order model, whereas lumped parameters such as α or α_H are treated as effective couplings within the corresponding formulation.

Table S2: Summary of lead-free candidate families discussed in the manuscript and ESI (qualitative; values and observations are those explicitly cited in the text).

Family	Examples (as cited)	Supporting observations reported in the cited literature
Lead-free double perovskites	$Cs_2AgBiBr_6$	Indirect bandgap ∼2.18 eV; hydrogenation can reduce bandgap to 1.7–1.9 eV; direct optical transitions near 2.1–2.2 eV; thin-film thickness 100–200 nm; porous nanoplatelet architectures; mobilities 1–10 $\mathrm{cm^2V^{-1}s^{-1}}$ and carrier lifetimes 18–42 ns; diffusion coefficient reference $D \sim 10^{-14}$–10^{-13} $\mathrm{cm^2s^{-1}}$; degradation under 85°C/85% RH; compatible with optical device configurations and composite thick films. [1,4–6]
Bi-based halide perovskite derivatives	$A_3Bi_2Br_9$ (A = Cs, MA, FA)	Vacancy-ordered 0D/2D motifs; stimulus-dependent optical shifts (thermochromism: band-gap decrease ∼0.13–0.14 eV from room temperature to 120°C; high-pressure response with reported trigonal-to-monoclinic transition in the ∼4.6–5.5 GPa range); violet/UV QD emission 379–400 nm; narrowband blue photodetection (e.g., ∼0.1 $\mathrm{mA\,W^{-1}}$ at 470 nm; spectral width as low as 24 nm); dual-band photodetection via $Cs_3Bi_2Br_9/Cs_3BiBr_6$ BHJ; radiation detection context for $Cs_3Bi_2Br_9$. [8–14]
Tin-based hybrid perovskites	$MASnI_3$, $FASnI_3$	Rapid oxidative degradation and air sensitivity (noted as potentially enabling fast responses when controlled degradation is the intended operating mode).

S3 Additional sensitivity analyses

These supplementary analyses assess the robustness of the main qualitative design conclusions with respect to optical nonlinearity, transport regime, kinetic uncertainty, and enzymatic saturation, while also indicating where quantitative performance becomes more sensitive to parameter variation.

S3.1 Sensitivity to the optical nonlinearity parameter β

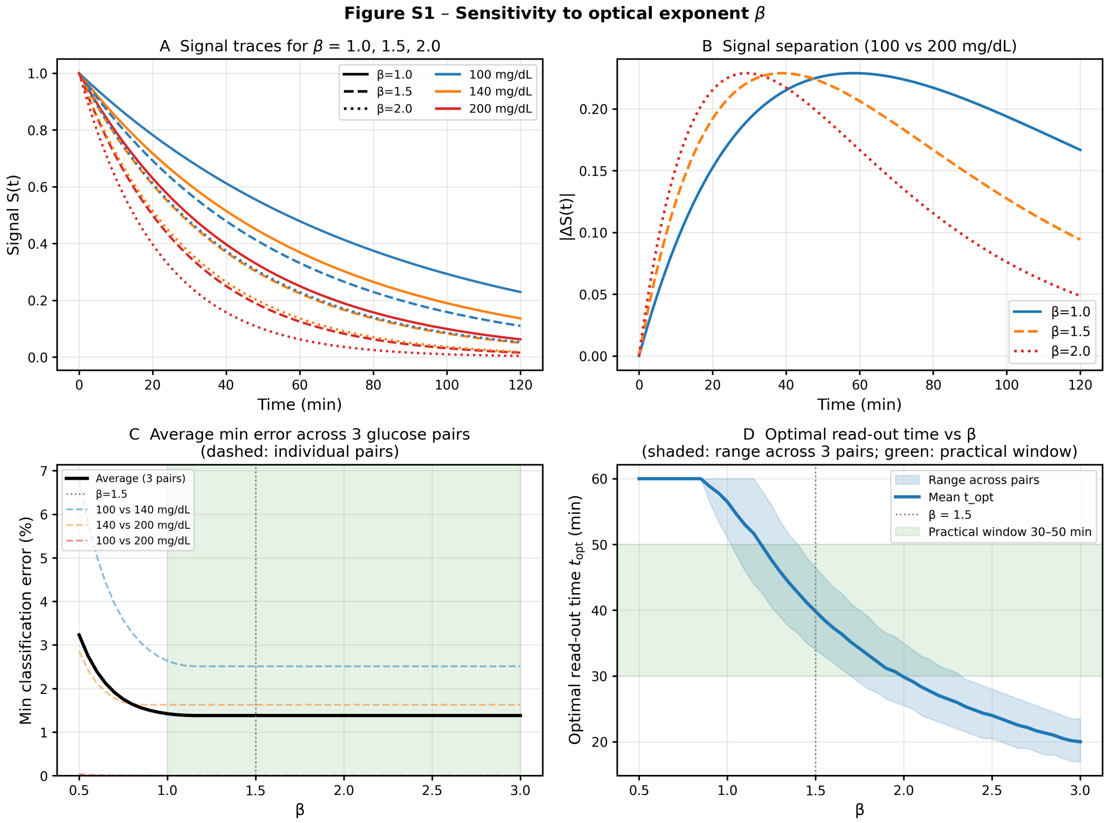

Figure S1: Sensitivity of the optical response to the empirical exponent β. (A) Representative signal trajectories for $\beta = 1.0$, 1.5, and 2.0. (B) Signal separation for the 100 vs 200 $\mathrm{mg\,dL^{-1}}$ pair. (C) Minimum classification error averaged over three representative glucose pairs (black), with individual pairs shown as dashed lines. The average error approaches a broad low-error plateau for intermediate-to-large β, so that further increases beyond this regime provide little improvement in discrimination. (D) Optimal read-out time t_{opt} (mean and range across pairs) versus β. The green band marks the practical 30–50 min window. Small β values shift t_{opt} to later times, whereas larger β values compress the response toward earlier read-out. In this sense, $\beta \approx 1.5$ represents a practical intermediate regime that combines near-minimal classification error with an operationally convenient read-out window for the representative cases explored here.

S3.2 Transport-regime sweep

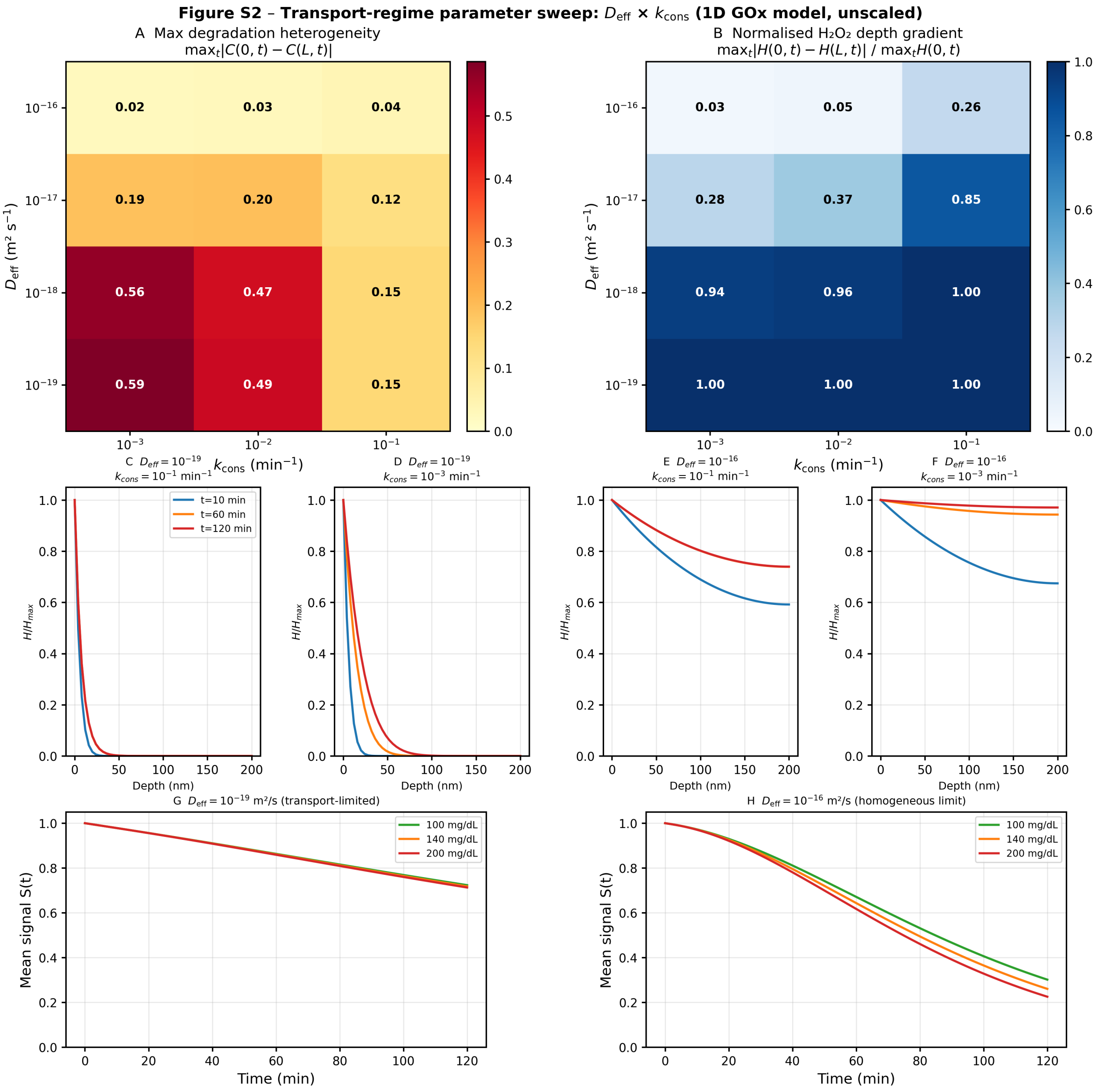

Figure S2: Transport-regime sweep over effective peroxide diffusivity D_H and peroxide-consumption rate k_{cons}. The map identifies the transition from strongly surface-localised degradation to the nearly homogeneous limit, and clarifies when a 1D transport-aware treatment becomes necessary.

S3.3 Robustness to kinetic uncertainty

To assess robustness to moderate kinetic uncertainty, we perturb the nominal reduced-order kinetic parameters directly rather than the prescribed half-lives. In panels A/C, the basal degradation rate is varied as

$$k_0 \to k_0(1+\delta)$$

while keeping k_G fixed. In panels B/D, the glucose-to-degradation coupling is varied as

$$k_G \to k_G(1+\delta)$$

while keeping k_0 fixed. The perturbation levels are $\delta = \pm 10\%, \pm 20\%, \pm 30\%$. Because, for first-order kinetics, $t_{1/2} = \ln 2/k$, these perturbations are related to corresponding half-life changes,

but they do not represent independent variation of the prescribed pair $(t_{1/2}^{(0)}, t_{1/2}^{(\mathrm{ref})})$.

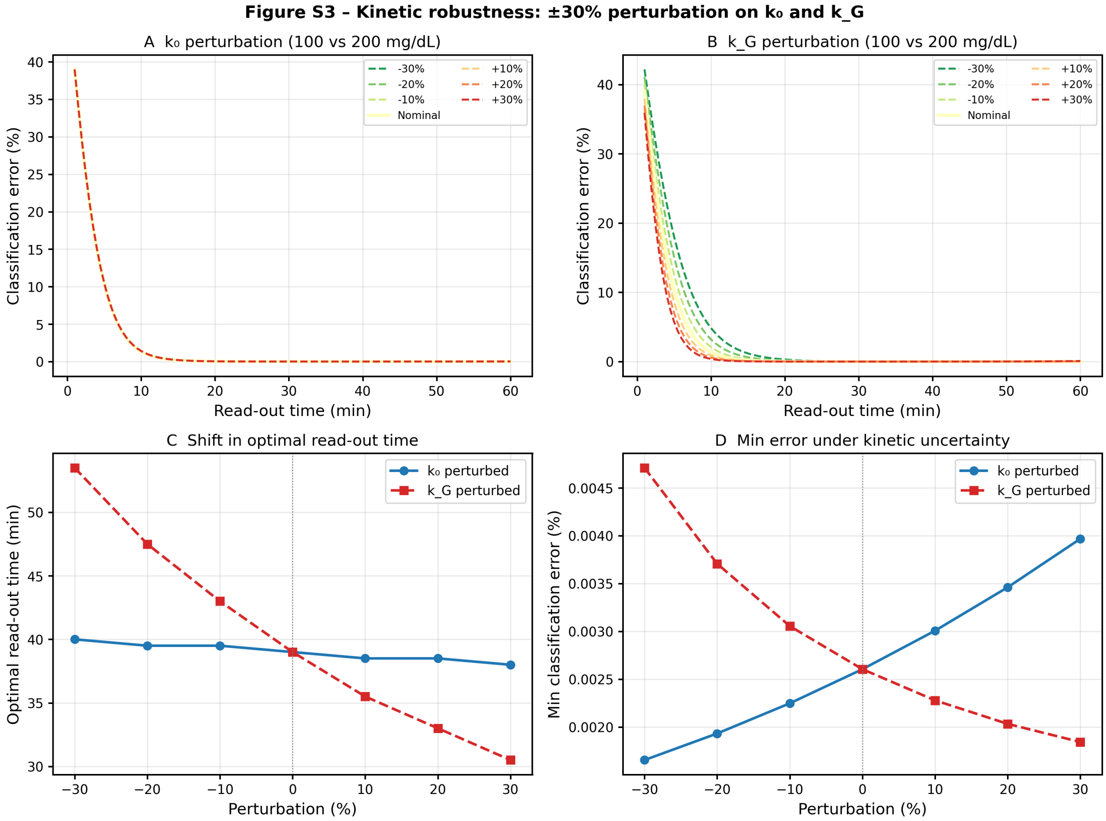

Figure S3: Robustness of the predicted read-out window and discrimination behaviour to moderate perturbations in the nominal kinetic parameters. Panels A/C perturb the basal degradation rate as $k_0(1+\delta)$ with k_G fixed, whereas panels B/D perturb the glucose-to-degradation coupling as $k_G(1+\delta)$ with k_0 fixed, using $\delta = \pm 10\%, \pm 20\%, \pm 30\%$. The practical operating window remains in the same approximate range under these parameter variations.

S3.4 Comparison between linearised and full Michaelis–Menten peroxide generation

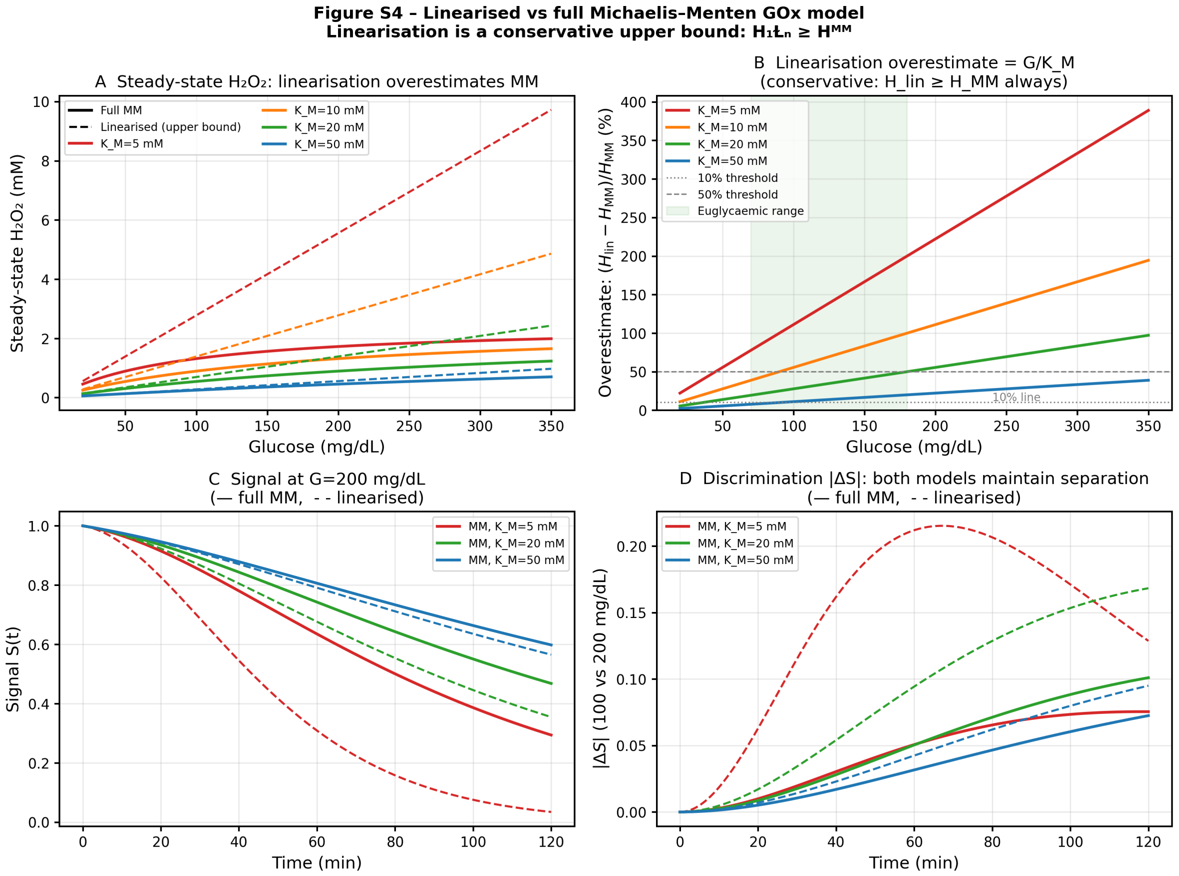

Figure S4: Comparison between the linearised glucose-to-peroxide mapping and the full Michaelis–Menten production law across representative K_M values. Nonlinear saturation modifies the transient dynamics and generally reduces degradation severity relative to the linearised approximation, while preserving monotonic signal separation across the explored range.

S3.5 Impact of Michaelis–Menten saturation on discrimination performance

To assess more directly whether nonlinear Michaelis–Menten saturation materially affects discrimination performance, we compare the GOx-mediated model against the linear surrogate across representative K_M values while keeping the same noise level ($\sigma_S = 0.02$) and practical read-out constraint ($t_{\text{read}} \leq 60$ min) used in the main design analysis. For comparability, the GOx-mediated model is area-matched at the reference level $G_{\text{ref}} = 200\ \text{mg}\,\text{dL}^{-1}$, so that the comparison isolates how saturation redistributes contrast across other glucose levels rather than reflecting an arbitrary difference in overall scale.

Figure S5 shows that Michaelis–Menten saturation can materially increase the minimum classification error, especially for closer glucose pairs, because saturation compresses the effective peroxide contrast between glucose levels. At the same time, the signal trajectories remain monotonic and the optimal read-out time stays within a practical tens-of-minutes range over the explored K_M values. The strongest sensitivity is observed for lower K_M values and for closer glucose pairs, whereas higher-contrast pairs remain more robust.

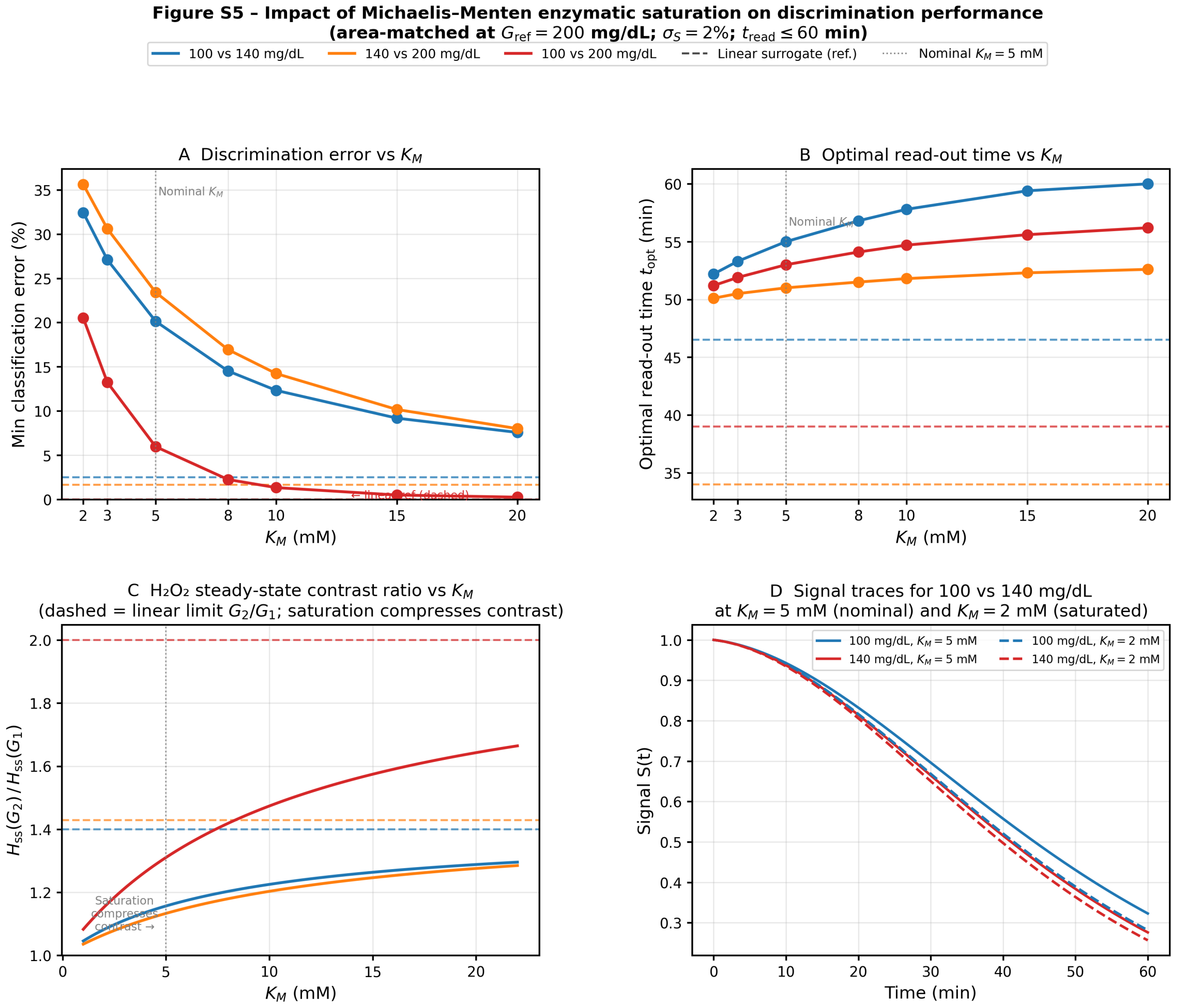

Figure S5: Impact of Michaelis–Menten enzymatic saturation on discrimination performance, using the GOx-mediated model area-matched at $G_{\mathrm{ref}} = 200\ \mathrm{mg\,dL^{-1}}$, with $\sigma_S = 2\%$ and $t_{\mathrm{read}} \leq 60$ min. (A) Minimum classification error as a function of K_M for representative glucose pairs, compared against the linear surrogate reference. (B) Corresponding optimal read-out time t_{opt} as a function of K_M. (C) Steady-state peroxide contrast ratio versus K_M, showing saturation-induced compression relative to the linear limit. (D) Representative signal traces for the 100 vs 140 $\mathrm{mg\,dL^{-1}}$ pair at nominal ($K_M = 5$ mM) and more strongly saturated ($K_M = 2$ mM) conditions. Nonlinear saturation increases classification error quantitatively, especially for closer glucose pairs, while preserving monotonic signal separation and a practical read-out window in the explored range.

References

[1] M. Sotelo-Lerma, L. Fernandez-Izquierdo, M. A. Ruiz-Molina, I. Borges-Doren, R. Haroldson and M. Quevedo-Lopez, *Materials*, 2024, **17**, 5123.

[2] A. F. Gualdrón-Reyes, R. Fernández-Climent, S. Masi, C. A. Mesa, C. Echeverría-Arrondo, F. Aiello, F. Balzano, G. Uccello-Barretta, J. Rodríguez-Pereira, S. Giménez and I. Mora-Seró, *Advanced Optical Materials*, 2023, **11**, 2203096.

[3] M. S. Pradeepkumar, A. Kathirvel, S. Ghosh *et al.*, *Scientific Reports*, 2025, **15**, 843.

[4] Z. Zhang, Q. Sun, Y. Lu *et al.*, *Nature Communications*, 2022, **13**, 3397.

[5] M. Mehrabian, M. Taleb-Abbasi and O. Akhavan, *Materials for Renewable and Sustainable Energy*, 2025, **14**, 38.

[6] Q. Chen, A. K. Singh, H. Alghathami, X. Liu, K. Huang, A. G. Thomas, R. J. Curry, L. J. Phillips and A. J. Hughes, *Green Chemistry*, 2025, **27**, 7532–7543.

[7] H. W. Lei, D. Hardy and F. Gao, *Advanced Functional Materials*, 2021, **31**, 2105898.

[8] M. Li, W. Li and H. Wei, *Advanced Photonics Research*, 2025, **6**, 2400095.

[9] N. K. Tailor, J. Kruszynska, D. Prochowicz, P. Yadav and S. Satapathi, *Advanced Optical Materials*, 2024, **12**, 2301583.

[10] Q. Li, L. Yin, Z. Chen, K. Deng, S. Luo, B. Zou, Z. Wang, J. Tang and Z. Quan, *Inorganic Chemistry*, 2019, **58**, 1621–1626.

[11] L. Wu, Y. Pan, Y. Zhang, Y. Geng, J. Cao, X. Su, J. Xu, H. Xie and D. Gao, *Advanced Functional Materials*, 2025, **35**, 2415315.

[12] Z.-C. Wu, J. Liu, X.-D. Lu, J. Wang, Z.-Y. Huang, Z.-G. Zhu, C.-C. Wang, Y. Wang, F.-X. Liang and L.-B. Luo, *IEEE Transactions on Electron Devices*, 2025, **72**, 1828–1832.

[13] F. Cao, Z. Li, X. Liu, Z. Shi and X. Fang, *Advanced Functional Materials*, 2022, **32**, 2206151.

[14] X. Li, X. Du, P. Zhang, Y. Hua, L. Liu, G. Niu, G. Zhang, J. Tang and X. Tao, *Science China Materials*, 2021, **64**, 1427–1436.

[15] S.-Y. Kim, D.-A. Park and N.-G. Park, *ACS Applied Electronic Materials*, 2022, **4**, 2388–2395.

[16] F. Zeng, Y. Tan, B. Sun, W. Hu, H. Yin, X. Tang, L. Huang, J. Liao and M. Tang, *ACS Omega*, 2025, **10**, 33731–33740.
